# Assisted Gene Flow Management to Climate Change in the Annual Legume *Lupinus angustifolius* L.: From Phenotype to Genotype

**DOI:** 10.1111/eva.70087

**Published:** 2025-03-06

**Authors:** S. Sacristán‐Bajo, C. Lara‐Romero, A. García‐Fernández, S. Prieto‐Benítez, J. Morente‐López, M. L. Rubio Teso, E. Torres, J. M. Iriondo

**Affiliations:** ^1^ Global Change Research Institute (IICG) Rey Juan Carlos University (URJC) Móstoles Madrid Spain; ^2^ Departamento de Medio Ambiente, CIEMAT Grupo de Ecotoxicología y Contaminación del Aire Madrid Spain; ^3^ Department of Plant Evolutionary Ecology Goethe‐University Frankfurt Frankfurt am Main Germany; ^4^ Departamento de Biotecnología‐Biología Vegetal Universidad Politécnica de Madrid Madrid Spain

**Keywords:** adaptive potential, assisted gene flow, climate change, evolutionary changes, flowering onset, genomics

## Abstract

Climate change may hinder species' ability to evolutionarily adapt to environmental shifts. Assisted gene flow, introducing adaptive alleles into target populations, could be a viable solution for keystone species. Our study aimed to evaluate the benefits and limitations of assisted gene flow in enhancing the evolutionary potential of *Lupinus angustifolius* L. (Fabaceae), considering both phenotypic and genomic perspectives. We collected seeds from four populations in Spain at two latitudes (north and south), and grew them in a common garden. We used pollen from southern individuals to pollinate northern plants and create an F1 gene flow line that would advance its flowering onset. In the next season, we allowed F1 plants to self‐pollinate creating an F2 self‐pollination line. We also created a backcross line by pollinating control northern plants with pollen from F1 plants. We measured flowering onset, reproductive success, and other plant traits in all resulting lines. In parallel, we sequenced genes related to reproduction, growth, stress, nitrogen, and alkaloids. All gene flow‐derived lines flowered significantly earlier than the control lines from the northern populations. F1 gene flow line plants produced heavier seeds and had a lower shoot growth than those from the northern control lines. Genomic analyses identified 36 outlier SNPs between the control and the F1 gene flow lines, associated with differences in flowering onset, seed weight, and shoot growth. These results underscore that assisted gene flow can enhance a population's evolutionary potential by altering specific traits. However, altering one trait may impact others in a way that depends on the intrinsic characteristics of each population.

## 1 Introduction

Accelerated climate change, driven by human activity, is threatening the survival of many species (IPCC 2022). Species can respond to this change through migration to more favorable areas, phenotypic plasticity, or evolutionary adaptation (Jump and Peñuelas 2005; Parmesan and Yohe 2003). However, effective migration may not be feasible for all organisms, and evolutionary adaptation is contingent on the genetic variation, demography, and historical processes of populations (Sheth and Angert 2016). In some instances, the pace of environmental change outstrips the rate at which species can adapt or migrate, leading to a climate‐response mismatch (Aitken and Whitlock 2013). Consequently, there is an increasing need for management strategies that enhance the adaptive potential of target populations, thereby mitigating extinction risks.

Assisted migration, a strategy involving the physical translocation of populations to areas outside the present range of the species predicted to be favorable under future climate scenarios, has been suggested as a potential solution (Aitken and Whitlock 2013; Grady et al. 2011; Loss et al. 2011; Vitt et al. 2010). However, this approach has sparked considerable debate due to the potential ecological risks. Concerns include major impacts on biotic communities, alterations to nutrient cycles, and disruptions to ecological processes such as pollination or seed dispersal (Mack et al. 2000; Traveset and Richardson 2006). There is also the risk of hybridization with other species, the possibility of the translocated species becoming invasive, and the inadvertent transfer of pathogens (Loss et al. 2011; Williams and Dumroese 2013). Furthermore, the impacts of these introductions may not be immediately apparent and can vary greatly over space and time (Ricciardi and Simberloff 2009).

Assisted gene flow is an alternative strategy that could address some of the challenges derived from assisted migration (Aitken and Whitlock 2013; Prieto‐Benítez et al. 2021; Torres et al. 2023; Wadgymar et al. 2015). It involves the transfer of gametes or individuals between existing populations to facilitate adaptation (Aitken and Whitlock 2013; Whiteley et al. 2015). Gene flow between populations is known to increase genetic variability, enabling adaptive responses to new scenarios such as climate change (Grummer et al. 2022). While the creation of corridors has been proposed to facilitate gene flow (Beier 2012; Heller and Zavaleta 2009), these connections are not always feasible. Moreover, natural gene flow is inherently limited in certain plant species, such as those lacking seed dispersal mechanisms or strictly autogamous plants. Thus, it is crucial to explore strategies to enhance the adaptation of populations and species, facilitating their survival.

Assisted gene flow has emerged as a promising tool in conservation and genetic management (Grummer et al. 2022). While its potential for enhancing the adaptive potential of populations to climate change remains underexplored, it offers several advantages over assisted migration. Unlike the latter, assisted gene flow involves transferring genes or individuals only between existing populations, thereby minimizing ecological risks (Aitken and Whitlock 2013). One key benefit of assisted gene flow is its broader geographical reach, as gametes can be transferred over vast distances. Furthermore, its directional nature increases the likelihood that introduced alleles are pre‐adapted to current and future environmental pressures. This contrasts with natural gene flow, which occurs indiscriminately and may lead to maladaptation (Aitken and Whitlock 2013). However, assisted gene flow can also prompt other genetic risks, such as outbreeding depression, genetic swamping, and a loss of local adaptation. Thus, some introduced alleles may struggle to adapt to the new conditions, resulting in reduced fitness (Aitken and Whitlock 2013; Byrne et al. 2011; Edmands 2007; Frankham et al. 2011; Grummer et al. 2022). Given these considerations, deepening our understanding of both the risks and benefits of assisted gene flow is crucial. This knowledge will help us better understand the evolutionary capacity of populations while also evaluating it as a tool to foster adaptation (Frankham et al. 2017).

Within a species, ecologically significant traits often vary along environmental gradients (de Frenne et al. 2013; Milla et al. 2009). Phenological traits, for instance, are closely related to climate conditions, with organisms constantly striving to align their phenologies with optimal environmental circumstances (Pau et al. 2011). Phenological shifts are, therefore, among the most notable impacts of climate change (Bradshaw and Holzapfel 2009; Parmesan and Yohe 2003), with flowering onset playing a crucial role in plant adaptation to climate change (Franks and Hoffmann 2012). Populations typically exhibit differences in flowering onset based on latitude, with lower latitude populations generally flowering earlier (Lévesque et al. 1997). Several studies have confirmed that flowering onset is a genetically controlled trait with high heritability (Riihimäki and Savolainen 2004; Franks et al. 2007; Méndez‐Vigo et al. 2013). Moreover, flowering onset is a polygenic trait involving numerous genes and complex regulation (Blümel et al. 2015; Fagny and Austerlitz 2021), although in some cases one locus can have a major effect (Wang et al. 2018). It is important to consider that the timing of flowering onset often correlates with other vital traits for plant survival and reproductive success, potentially constraining its evolution (Etterson and Shaw 2001; Sacristán‐Bajo et al. 2023; Walsh and Blows 2009). Therefore, a better understanding of the genomic basis of flowering onset and potential genetic constraints due to trait correlations could help in the design of future assisted gene flow actions.

Given the limited evidence available on assisted gene flow, this study aimed to explore its potential for advancing flowering onset in plant populations, while carefully assessing the associated risks. We conducted an experimental study with *Lupinus angustifolius* L. (Fabaceae), an autogamous annual plant species. This study involved manual crosses between four populations from two climatically distinct areas of the Iberian Peninsula, and the phenotypic and genotypic characterization of the progeny in a common garden environment. A previous common garden experiment with these populations found that the southernmost populations (with warmer climate patterns) flower earlier than the northernmost populations (Sacristán‐Bajo et al. 2023). Therefore, we anticipated that gene flow from southern to northern populations would advance flowering onset in the offspring with respect to average northern population individuals. Given the genetic distinctiveness of the southern populations used as sources for gene flow (Sacristán‐Bajo et al. 2023), we hypothesized that the introduction of new alleles into northern populations would also alter other traits. We expected the first gene flow generation to produce hybrids with intermediate phenotypes. The second generation, produced by self‐fertilization of the hybrids, would exhibit trait segregation, resulting in diverse phenotypes. Finally, the backcross line, obtained by the subsequent pollination of the hybrids with individuals from the northern populations, would also exhibit trait segregation but with patterns more closely related to the northern populations. We also hypothesized that a genomic signature associated with the expected phenotypic changes could be identified. To test these hypotheses, we recorded the timing of flowering onset and other plant traits (plant height, biomass, shoot growth, seed number and weight, specific leaflet area or SLA and leaflet dry matter content or LDMC), and sequenced genes related to the flowering process and abiotic stress that may affect these traits. We then compared the gene flow lines' results against those of the control line to answer the following questions: (i) Can the flowering of northern populations be advanced through assisted gene flow from southern populations? (ii) If so, are there other traits of the individuals modified with the gene flow? (iii) What is the genomic signature of the phenotypic changes brought about by the gene flow lines?

## 2 Materials and Methods

### 2.1 Study Species and Source Populations

The blue lupine ( *Lupinus angustifolius* ) is an annual legume native to the Mediterranean basin. This plant can grow over 100 cm tall and features characteristic palmate leaves divided into 5–9 leaflets. Its hermaphroditic flowers form inflorescences of up to 30 flowers. The fruit is a dehiscent legume with 3–7 seeds (Clements et al. 2005). The species primarily self‐pollinates before its petals open (Wolko et al. 2011), with outcrossing estimates below 2% (Dracup and Thomson 2000). Flowering occurs between March and August, depending on latitude and environmental conditions, as its flowering onset is influenced by photoperiod and temperature (Castroviejo and Pascual 1993; Rahman and Gladstones 1974). This plant has been domesticated as a crop and is cultivated worldwide (Castroviejo and Pascual 1993).

**FIGURE 1:**
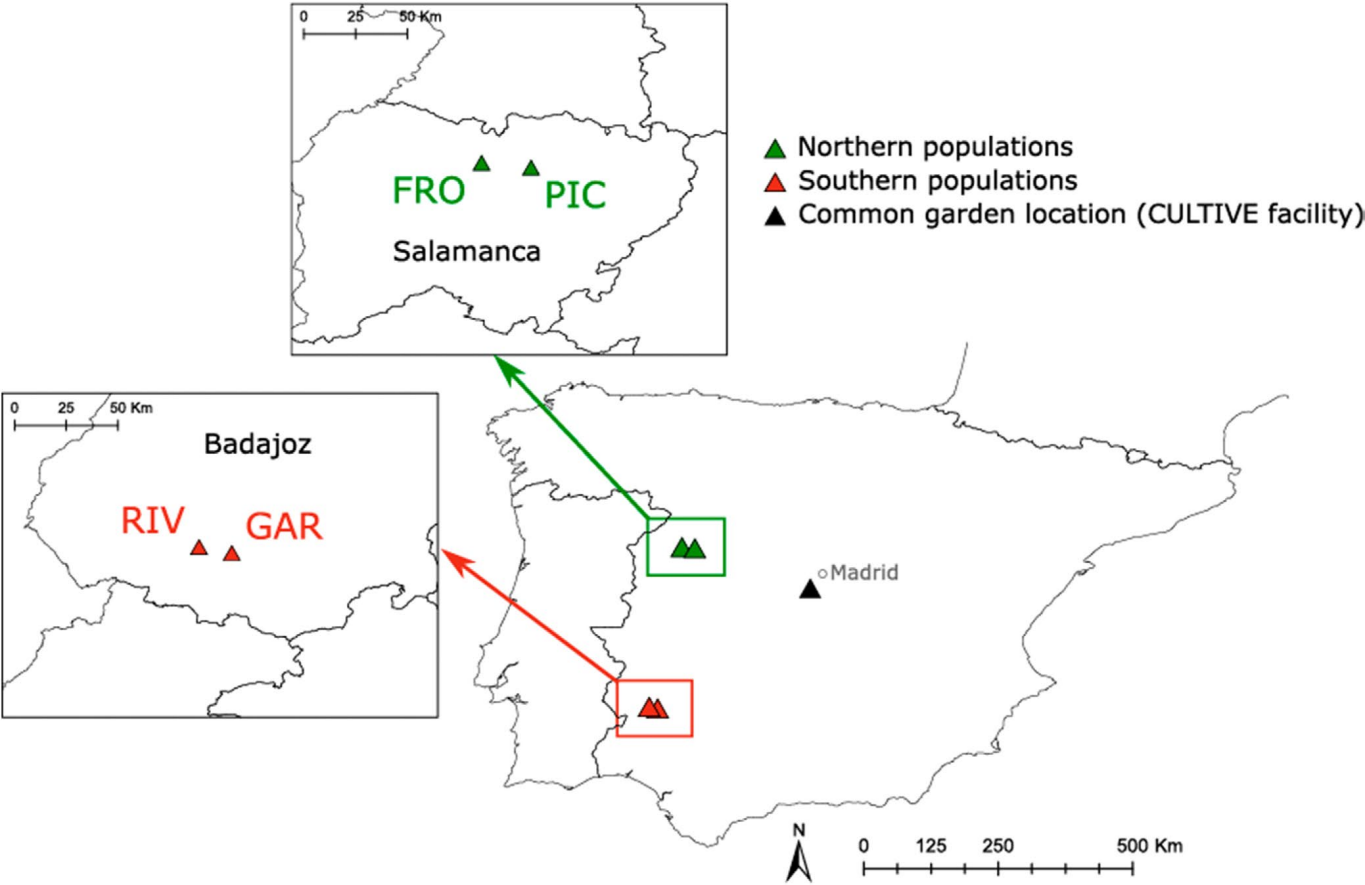
Location of northern (green) and southern (pink) populations of *Lupinus angustifolius* L. and the common garden established in the Iberian Peninsula.

For our study, we selected four populations distributed by pairs from two climatically contrasting regions in Spain: Salamanca in Central Spain (northern populations) and Badajoz in Southern Spain (southern populations) (Figure 1, Table 1). The regions are approximately 300 km apart, with less than 20 km between the populations within each region. Both regions have similar annual precipitation, but the southern region has markedly lower May–July precipitation and higher mean, minimum, and maximum temperatures, leading to greater water deficits. In each population, we collected seeds from at least 98 genotypes (mother plants), each located at least 1 m apart.

**TABLE 1:** Populations of *Lupinus angustifolius* L. and common garden site involved in the study. Acronym, town, region, geographical coordinates (decimal degrees, WGS84), and climate variables associated to the populations (1985–2015 period) and to the common garden site (average of the years 2017–2020). May–July period corresponds with the period when the plants are developing fruits and setting seeds. Climate data were obtained from ClimateEU (Marchi et al. 2020).

Acronym	Town	Region	Latitude	Longitude	Elevation (m. a.s.l.)	Annual mean temperature (^o^C) and coefficient of variation (in brackets)	May–July precipitation (mm) and coefficient of variation (in brackets)
FRO	Zafrón	Northern Spain	41.0241	−6.0281	840	12.4 (3.2)	92 (46)
PIC	Zarapicos	Northern Spain	41.0043	−5.8130	820	12.6 (3.1)	89 (45)
GAR	La Garranchosa	Southern Spain	38.3257	−6.4337	422	16.5 (2.4)	64 (64)
RIV	Rivera de la Lanchita	Southern Spain	38.3515	−6.5760	352	16.8 (2.2)	61 (63)
—	Common garden (2017–2020)	Central Spain	40.3343	−3.8829	690	14.9	63

### 2.2 Gene Flow Experiment

**FIGURE 2:**
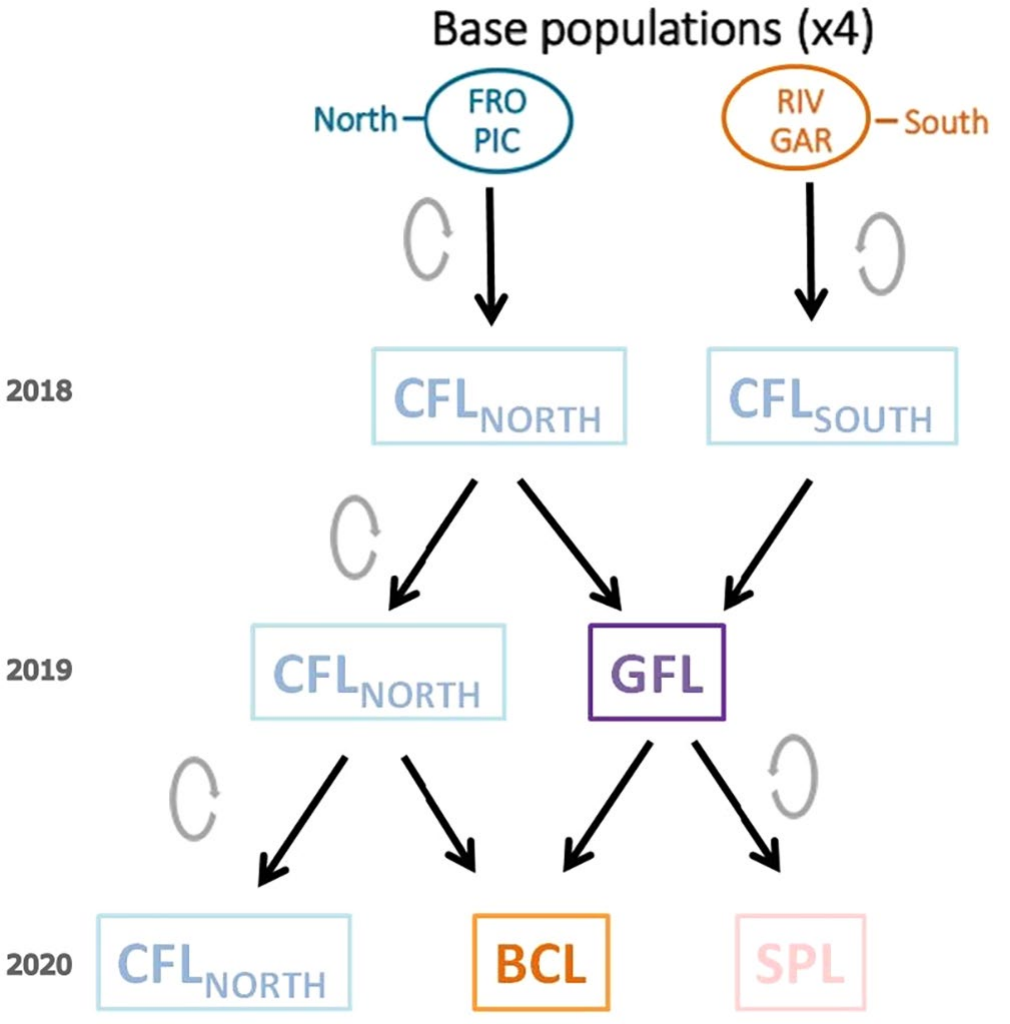
Flowchart describing the different lines obtained with individuals of four populations of *Lupinus angustifolius* through time. CFL, control line; GFL, F1 gene flow line; BCL, F2 backcross line; SPL, F2 self‐pollination line. Gray circled arrows indicate that the individuals of that line were self‐crossed. Black arrows denote the transmission of gametes for the next generation. The years indicated on the left correspond to the flowering season in which adult plants of the indicated lines had been grown and the crosses performed in that season are represented below with the arrows.

The common garden experiment was conducted at the CULTIVE facility (https://urjc‐cultive.webnode.es/) at Rey Juan Carlos University (Móstoles, Madrid). In November 2016, 12 seeds from 22 randomly selected maternal genotypes per population were scarified to ensure germination and sown in groups of three in four 6 L pots (3 seeds per pot, 4 pots per genotype), following the same protocol described in Sacristán‐Bajo et al. (2023). The temperature ranged from 1°C to 25°C, and plants received only natural light. In spring 2017, the pots were transferred outside of the greenhouse to the CULTIVE experimental field and arranged in a randomized block design, with plants from the different populations evenly represented in each block. The substrate in the pots was kept at field capacity with a drip irrigation system. The temperature conditions at this site are intermediate between those found at the northern and southern regions of origin (Table 1). Before flowering, main‐stem inflorescences were bagged to obtain seeds derived from self‐pollination. For each population, their seeds were separately collected to generate the corresponding “control lines” (CFL_NORTH_ and CFL_SOUTH_, Figure 2). This first growing season was used solely to eliminate maternal effects.

In November 2017, seeds collected separately from each individual were sown under the same conditions as described above, and the resulting plants were transferred to the CULTIVE experimental field in February 2018. During the 2018 flowering season, the control lines of each northern population were self‐pollinated to generate the control lines of the 2018–2019 season (Figure 2). In addition, manual between‐population crosses were carried out to create an “F1 gene flow line” (GFL, Figure 2). Plants from the northern region were pollinated using pollen from plants from the southern region, matching the RIV population with the PIC population and the GAR population with the FRO population (Figure 2). All possible crosses between these two pairs of populations (considered as replicates) were performed considering that overlapping flowering periods between their individuals were needed. The procedure to carry out manual crosses was the same as that described in the Supporting Information of Sacristán‐Bajo et al. (2023), based on the emasculation of individuals of the northern region and their subsequent pollination with pollen from individuals from the southern region.

Seeds produced were collected separately for each mother plant. In the 2018–2019 season, seeds were sown, and seedlings were cultured and transferred outdoors in the same way as described above, containing, for each population, individuals from the CFL_NORTH_ and GFL lines. In the 2019 flowering season, the CFL_NORTH_ individuals of the corresponding northern populations were manually pollinated using GFL individuals as pollen donors, creating a “backcross line” (BCL, Figure 2). Additionally, an “F2 self‐pollination line” (SPL, Figure 2) from the GFL was generated by self‐pollination. Seeds from the CFL_NORTH_ were self‐crossed to maintain the control line, thus forming the CFL_NORTH_ of the 2019–2020 season (Figure 2). The seeds of these lines were again separately collected for each mother plant. In the 2019–2020 season, the seeds from these lines were sown and the resulting seedlings were grown and transferred outdoors as indicated above. A diagram of the complete process is shown in Figure 2. Results of the backcross line (BCL) are only shown for the FRO population since manual crosses were not successful, and therefore, it was not possible to obtain seeds for the PIC population.

### 2.3 Traits Measurement

The day of flowering onset was recorded for each plant as the day when the first purple flower of the main inflorescence was clearly visible and calculated as the number of days between sowing and flowering start date. We estimated the number of fruits per plant based on the total number of floral scars at the end of the season. The average number of seeds per fruit was determined by counting the seeds in 15 different fruits per plant. The number of seeds per plant was calculated by multiplying the number of fruits per plant by the average number of seeds per fruit. The individual weights of 10 random seeds from each plant were used to calculate the mean seed weight. The mean seed weight and the number of seeds per plant were used as proxies for determining plant fitness.

We also estimated the height of the plants (cm) at the flowering peak by measuring the distance from the ground level to the base of the main inflorescence. At the start of flowering and at the end of the culture cycle, we measured the length of the plant (cm) from its base to the first flower. The difference between these two values was used to estimate the shoot growth (cm) of each individual. We also weighed the aboveground biomass (g) of each plant at the end of the culture cycle. The central leaflet from eight fully developed leaves belonging to the lateral branches was gathered to determine the specific leaflet area (SLA) and dry matter content (LDMC). The fresh leaflets were weighed immediately on a Kern ABJ 120‐4 M analytical balance (Kern & Sohn GmbH, Albstadt, Germany), then placed in water‐soaked filter paper and stored in plastic bags before being refrigerated overnight at 4°C. We weighed the leaflets again the next day to get the turgid weight and used a foliar scanner Li‐3000C (Li‐Cor, NE, United States) to measure the area of the leaflets. Finally, the leaflets were dried for at least 72 h in a 60°C oven before being weighed again to determine their dry weight. SLA was calculated by dividing the area of a leaflet by its dry weight (Rosbakh et al. 2015). LDMC was determined by dividing the leaflets' dry weight by its saturated weight (Wilson et al. 1999).

The flowering onset was measured for the years 2019 and 2020, but due to the mobility restrictions of the pandemic lockdown, the rest of the traits were only measured for the year 2019.

### 2.4 Phenotypic Analyses

All statistical analyses were conducted using the R statistical environment version 4.1.1 (R Core Team 2020). We applied linear and generalized linear mixed models (LMMs and GLMMs) to analyze the effect of the F1 gene flow line, the F2 self‐pollination line, and the backcross line on flowering onset and other traits. For each trait, we included the *line* (CFL_NORTH_2019_ and GFL for the year 2019, and CFL_NORTH_2020_, SPL and BCL for the year 2020) and the *population* (FRO and PIC) as fixed effects, and *genotype* (mother plant) as a random effect. Diagnostic plots were used to visually confirm the normality and variance homogeneity of model residuals for normality. The R package DHARMa (Hartig 2022) was used for this purpose. Since the flowering onset variable holds count data, we used a Poisson error distribution (GLMMs). For the remaining variables, we used a Gaussian error distribution (LMMs). We tested the interaction between *line* and *population* variables. As the interaction was not significant, it was not included in the models. The *glmer* and *lmer* functions from the *lme4* package version 1.1–27.1 were used to fit the GLMMs and LMMs (Bates et al. 2015). The *Anova* function from the *car* package version 3.0–11 was used to determine the significance of each fixed effect (Fox and Weisberg 2011). If necessary (as for the flowering onset in 2020), Tukey *post hoc* analysis from the *emmeans* function from the *emmeans* package version 1.6.3 was used to calculate differences between lines (Lenth 2019). *R* ^2^ values were calculated using the *summ* function from the *jtools* package version 2.2.0 (Long 2019). The *corrplot* function version 0.90 from the *corrplot* package was used to plot correlations between flowering onset and the other traits (Wei et al. 2017).

### 2.5 Genomic Analyses

#### 2.5.1 DNA Extraction and Selection of Candidate Genes

In 2019, leaf material was collected for DNA extraction from individuals of CFL_NORTH_2019_ and GFL lines that were also phenotyped. Leaves from a total of 60 individuals were collected, with 30 from each line (CFL_NORTH_2019_ and GFL) and 15 from each population (FRO and PIC) within each line. DNA was extracted and isolated using the DNeasy Plant minikit (QIAGEN, Valencia, USA).

We designed a gene capture experiment using the annotated *L. angustifolius* genome (Tanjil cultivar) from the National Center for Biotechnology Information (NCBI) as a reference (GenBank accession: PRJNA398717). This genome contains all coding sequences (CDs) belonging to the *L. angustifolius* genome. FullLengthNext software (Lara et al. 2007) and the *L. angustifolius* genome were used to perform a Blast analysis and obtain the biological function (i.e., gene ontology terms) for each sequence in *L. angustifolius* . We selected 73 gene ontology terms related to reproduction, growth, stress, nitrogen utilization, and alkaloids. After that, we used the Go.db R package to create a list with all the gene ontology terms superior (broader) and inferior (more specific) (i.e., parent and children Gene Ontology Terms following the convention used for describing relationships between GO Terms). Finally, we filtered the file containing all coding sequences to obtain the candidate genes of interest, based on the list of gene ontology terms. These sequences were used as probes to carry out the targeted sequencing.

#### 2.5.2 Sequencing and Single Nucleotide Polymorphism (SNP) Calling

The extracted DNA was sent to IGATech (Udine, Italy). The quality of the genomic DNA was checked using the Qubit 2.0 Fluorometer (Invitrogen, Carlsbad, CA) and the NanoDrop 1000 Spectrophotometer (Thermo Fisher Scientific, Waltham, Massachusetts). Libraries for target enrichment of ~3 Mb of *L. angustifolius* genomic material were produced using the ‘SeqCap EZ—HyperPlus’ kit (Roche Sequencing Solutions, Pleasanton, CA) with 200 ng/L of input DNA.

After that, base calling and demultiplexing were carried out with Illumina bcl2fastq v2.20. ERNE v1.4.6 (del Fabbro et al. 2013) and Cutadapt (Martin 2011) software were used for quality and adapter trimming; BWA‐MEM v0.7.17 (Li and Durbin 2009) for the alignment to the reference genome, and Picard tools (http://broadinstitute.github.io/picard/) to produce on‐target alignment statistics and metrics.

SNP calling was performed on the entire sample simultaneously with gatk‐4.0 (Depristo et al. 2011). This step allowed the initial identification of ca. 41,419 SNPs. Raw SNP data were filtered using VCFtools v0.1.14 (Danecek et al. 2011), and the *vcffilter* function of VCFLIB (Garrison et al. 2022). Only biallelic SNPs with fewer than 10% missing data were kept. Indels were also removed from the dataset. SNPs were then filtered following the hard filtering suggested by GATK's user guide (https://gatk.broadinstitute.org/). Hence, SNPs were filtered based on: (i) their quality depth (QD > 2), (ii) Phred scaled P‐value using Fisher's exact test to detect strand bias (FS < 60), (iii) Symmetric Odds Ratio of 2 × 2 contingency table to detect strand bias (SOR < 3), (iv) square root of the average of the squares of the mapping qualities (MQ > 40), (v) *z*‐score from Wilcoxon rank sum test of Alt vs. Ref read mapping qualities (MQRankSum> −12.5), (vi) *u*‐based *z*‐approximation from the Rank Sum Test for site position within reads (ReadPosRankSum> − 8) and (vii) depth coverage (DP > 10). This stringent filtering reduced the SNP dataset to 34,026 SNPs. Finally, SNPs in high linkage disequilibrium were filtered using *r* ^2^ of 0.6 as the cut‐off point, which generated a final dataset of 22,802 SNPs.

#### 2.5.3 Detecting Signatures of Selection

We applied a sequential strategy to identify highly divergent loci between the CFL_NORTH_2019_ and the GFL lines. We first calculated allele frequency differences (AFDs) between the CFL_NORTH_2019_ and the GFL at the individual SNP level and selected those SNPs that had experienced an allele frequency change in the same direction in both populations (FRO and PIC). We then selected those SNPs with significant AFDs by applying a Fishers's exact test (Fisher 1970). Secondly, pairwise F_ST_ values (CFL_NORTH_2019_ vs. GFL) were calculated for each SNP. Statistical significance of F_ST_ values was tested for each locus by the chi‐square test, *x* ^2^ = 2NF_ST_(*k*−1), with (*k*−1)(*s*−1) degrees of freedom, where *N* is the total sample size, *k* is the number of alleles per locus, and *s* is the number of populations (Workman and Niswander 1970). We only considered that an SNP showed divergent patterns of differentiation when it was selected as an outlier by both *F*_ST_ analyses and at the same time it showed consistent AFDs in the two pairs of CFL_NORTH_2019_ vs. GFL comparisons. Lastly, these highly divergent loci underwent an individual genotype–phenotype validation (Chen et al. 2022). For this purpose, a linear mixed model with random family effects was fitted using flowering onset, seed weight, and shoot growth as dependent variables, the genotype of each SNP as a three‐level explanatory factor (homozygous for the minor allele, homozygous for the major allele and heterozygous), individual as a random factor and a kinship matrix as a random genetic effect to control for kinship effects. We also included line (CFL vs GFL) as a fixed factor to minimize the effects of population structure as a confounding factor. This validation allowed us to detect those SNPs with a large effect on the phenotype. *F*_ST_ values and allele frequencies were calculated using VCFtools v0.1.14. Kinship matrix was calculated using the centered‐IBS method implemented in TASSEL v5.2.81 (Bradbury et al. 2007). Linear mixed models were fitted using the *lmekin* function implemented in *coxme* R package (Therneau 2020).

## 3 Results

### 3.1 Flowering Onset

**FIGURE 3:**
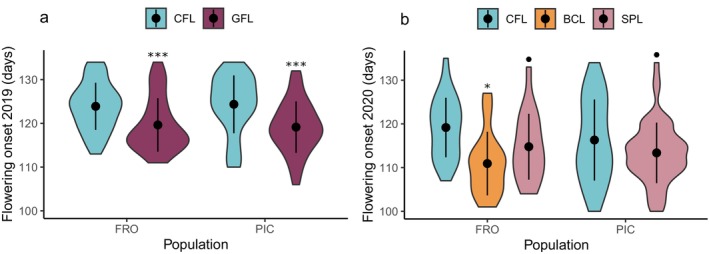
Effect of the gene flow line F1, self‐pollination line, and backcross line on advancing flowering onset of *Lupinus angustifolius* in 2019 (a) and 2020 (b). CFL, control line; GFL, F1 gene flow line; BCL, F2 backcross line; SPL, F2 self‐pollination line. Dots and bars represent the mean and 95% confidence intervals, respectively. Significant differences (*p* < 0.05) and marginally significant differences (*p* < 0.10) determined by Tukey test between the created lines and the control line are marked (*p* < 0.10; *p < 0.05; **p < 0.01; ***p < 0.001).

Significant differences in flowering onset were found between the gene flow lines and the control lines of the northern populations in 2019 (Figure 3a, Tables S2 and S3). In 2019, plants from the gene flow lines flowered an average of 7 days earlier than control plants in the FRO population and an average of 8 days earlier in the PIC population (*X* ^2^ = 17.42, *p* < 0.001, Df = 1) (Figure 3a, Table S1). In 2020, significant differences were also observed between the F2 self‐pollination lines and the control lines of the northern populations (*X* ^2^ = 6.96, *p* = 0.031, Df = 2) (Figure 3b, Tables S2 and S3). In 2020, the backcross line flowered 12 days earlier than the control line in the FRO population (Figure 3b, Table S1). The fixed effects explained 12.2% of the variation, and the random effects explained 1.8% of the variation in 2019. In 2020, fixed effects explained 16.6% of the variation, whereas the random effects explained 9.9% (Table S2). Table S4 shows the posterior mean values, standard errors, and 95% confidence intervals for each line.

### 3.2 Reproductive Success

**FIGURE 4:**
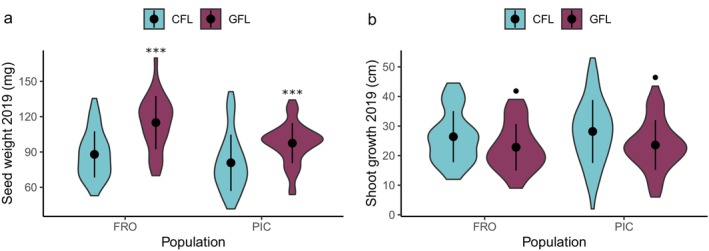
Effect of the gene flow line on the seed weight and shoot growth of *Lupinus angustifolius* in 2019 (a) and 2020 (b). CFL, control line; GFL, F1 gene flow line; BCL, F2 backcross line; SPL, F2 self‐pollination line. Dots and bars represent the mean and 95% confidence intervals, respectively. Significant differences (*p* < 0.05) and marginally significant differences (*p* < 0.10) determined by Tukey test between the created lines and the control line are marked (*p* < 0.10; *p < 0.05; **p < 0.01; ***p < 0.001).

In 2019, no significant differences were found between the gene flow lines and the control lines of the northern populations for seed number per plant (*X* ^2^ = 2.18, *p* = 0.140, Df = 1) (Figure S1a, Table S3). However, significant differences were obtained for seed weight (*X* ^2^ = 25.28, *p* < 0.001, Df = 1), where the seeds of the gene flow lines were heavier (Figure 4a, Tables S1, S2 and S3). Fixed effects accounted for 6.8% of the variation in seed number and 31% in seed weight, whereas random effects explained 6.9% and 15.6%, respectively. Posterior mean values, standard errors, and 95% confidence intervals for each line are shown in Table S4.

### 3.3 Vegetative Traits

Regarding height, biomass, SLA, and LDMC, no significant differences were observed between the control line and any of the established lines (Table S2, Figure S1). The only marginally significant difference between the gene flow lines and the control lines of the northern populations was in shoot growth (*X* ^2^ = 3.46, *p* = 0.06, Df = 1), with plants from the gene flow lines exhibiting lower shoot growth (Figure 4b, Tables S1, S2 and S3). The proportion of variation explained by fixed effects ranged from 2.2% to 10.3%, and by random effects from 0.2% to 40.2% depending on the trait (Table S2). Posterior mean values, standard errors, and 95% confidence intervals for each line are shown in Table S4.

### 3.4 Flowering Onset Correlations

In 2019, the control lines of the northern populations exhibited varying correlations between flowering onset and other plant traits (Figure S2). Notably, these correlations differed by population. For the FRO population, earlier flowering was associated with increased height (*r* = −0.33), biomass (*r* = −0.39), and seed weight (*r* = −0.41), but decreased shoot growth (*r* = 0.62) (Figure S2a). Conversely, in the PIC population, earlier flowering correlated with increased seed weight (*r* = −0.57) and biomass (*r* = −0.14), but reduced height (*r* = 0.33) and shoot growth (*r* = 0.92) (Figure S2b).

### 3.5 Loci Under Selection

**FIGURE 5:**
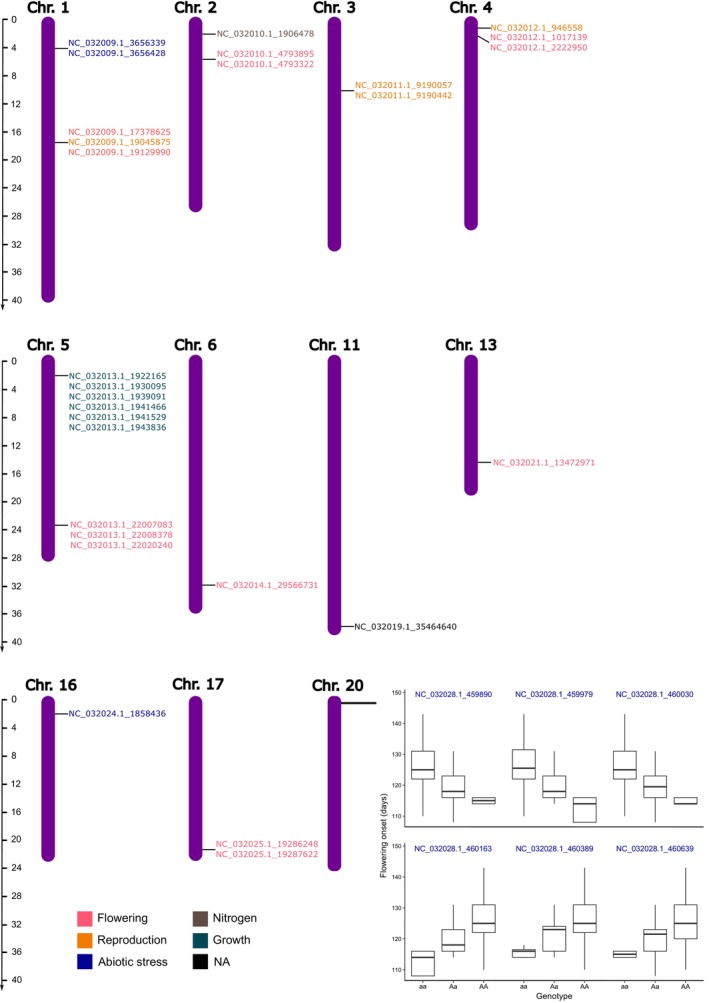
Localization of the SNPs identified to be under selection and with a significant effect on flowering onset, seed weight, and shoot growth on the chromosomes of *Lupinus angustifolius* and detail of the SNPs localized in chromosome 20 as an example. The name of the NCBI Reference Sequence and the position of the SNP in number of base pairs are indicated separated by a low bar. SNPs NW_017722081.1_3838 and NW_017722081.1_4164 are not shown in the figure because they could not be mapped on the chromosomes.

We identified 36 SNPs exhibiting divergent differentiation patterns, as they were outliers in F_ST_ analyses and exhibited consistent AFDs in control versus gene flow comparisons (Table S5). After controlling for line and kinship, these SNPs significantly affected flowering onset, seed weight, and shoot growth (Figures S3, S4 and S5) and displayed substantial allele frequency change between the control lines of the northern populations and the gene flow lines (Figure S6). The 36 significant SNPs were distributed across 11 of the 20 *L. angustifolius* chromosomes (Figure 5). Functional annotation revealed that these SNPs were associated with different biological processes. Six were related to reproduction, 6 to growth, 9 to abiotic stresses, and 13 to flowering, including the Flowering Locus T, EBS, and YABBY‐1 like proteins (See Table S6).

## 4 Discussion

Assisted gene flow resulted in significant advancements in flowering time for both *Lupinus angustifolius* populations. This intervention also affected seed weight and shoot growth. The genomic analysis revealed 36 SNPs with notable frequency differences between the gene flow lines and the control lines in both populations. These SNPs significantly accounted for variation in flowering onset, seed weight, and shoot growth, corroborating the genetic underpinnings of flowering advancement and the detected phenotypic‐level genetic correlations between flowering onset, seed weight, and shoot growth. These findings underscore the need for an integrative approach to assessing gene flow effects and demonstrate its potential as a tool for conservation.

### 4.1 Effects of Artificial Gene Flow on Plant Phenology, Reproductive Success, and Non‐Reproductive Traits

In Sacristán‐Bajo et al. (2023), we showed that southern populations flower earlier than northern ones under a common garden conditions. Consequently, we anticipated that hybrids from artificial crosses of northern mother plants with southern population pollen (GFL) would flower earlier than their respective northern controls. These results align with Bontrager and Angert's (2019) findings that gene flow from historically warmer populations of *Clarkia pulchella* enhanced the adaptive responses of colder populations amidst rising temperatures. However, contrary to our results, Prieto‐Benítez et al. (2021) observed a delay in flowering onset in recipient populations of *Silene ciliata* following gene flow from earlier‐flowering populations. This underscores the complexity and context dependency of assisted gene flow effects on flowering onset.

Epistatic effects between genes could render gene flow unpredictable or less effective (Blümel et al. 2015; He et al. 2019; Prieto‐Benítez et al. 2021), potentially explaining the greater flowering advance of the backcross line with regard to the gene flow line. This could be due to the mitigation of the adverse epistatic gene flow effects in the first generation with increased genome representation from the original (northern) populations. The possibility of carrying out successive generations of backcrossing with the northern populations while selecting early‐flowering progeny should be considered as a strategy to obtain individuals with the desired trait while recovering the original genome of the northern population. This would enable a safe reintroduction of these individuals into their target populations. On the other hand, the assisted gene flow did not seem to have caused outbreeding depression because no significant differences were found between the gene flow lines and the control lines of the northern populations for seed number per plant. In fact, some studies have shown that the implementation of assisted gene flow can be beneficial in the long term (Frankham 2015; Robinson et al. 2020).

Gene flow may not only shift the trait of interest (flowering onset in this case) but also induce changes in other traits, including those related to reproductive success (Aitken and Whitlock 2013; Morente‐López et al. 2021; Prieto‐Benítez et al. 2021). The heavier seeds obtained in the gene flow line (Figure 4a) might be interpreted as a result of heterosis in a predominantly selfing plant; however, this was not the case because the hybrids had simply an intermediate value between those of the northern and southern population individuals (Table S1).

Our study found that the gene flow lines exhibited lower shoot growth and a tendency towards lower SLA than the control lines of the northern populations. Correlation analyses between the studied traits also support these associations between flowering onset, seed weight, and shoot growth. For both populations, flowering onset correlated in the same direction for these traits (negatively with seed weight and positively with shoot growth). This suggests that early flowering plants have higher seed weight and lower shoot growth. In addition, these changes have also been observed at the genomic level (see next section). Several studies have shown that gene flow leads to changes in different plant traits. For example, Chacón‐Sánchez et al. (2021) reviewed the effects of gene flow between cultivated and wild types of several species of the genus *Phaseolus* (Leguminosae). Morphological, seed, and other traits were influenced by gene flow events and had important consequences for the species performance. In the context of climate change, shifts towards traits more similar to plants from the southern areas may confer an adaptive advantage. In line with our findings, Matesanz et al. (2020) noted that southern populations and those exposed to drought treatment had higher seed weight, lower growth rate, and thicker leaves. Given the limited plant resources, resource allocation for one purpose precludes its use for others (Reich 2014). Thus, the production of heavier seeds could enhance survival in harsher environments, such as the drier southern sites (Leishman et al. 2000; Metz et al. 2010), whereas lower shoot growth and lower SLA could indicate a more efficient resource investment (Wright et al. 1994). These results, along with others, reinforce the idea that gene flow‐induced modification of specific target traits will come along with changes in other traits due to the intricate links among biological traits and underscore the importance of interpreting the phenotype of the organism as a whole (Sobral 2021).

### 4.2 Genomic Effects of the Artificial Gene Flow

The integration of genome‐wide studies with phenotypic characterization is essential for identifying regions associated with adaptive variation (Evans et al. 2014). Our study identified 36 highly divergent SNPs between the control lines of the northern populations and the gene flow line, as indicated by both F_ST_ and AFD analyses, suggesting that these SNPs underwent genomic changes due to assisted gene flow. We also found that these SNPs partially explained variations in flowering onset, shoot growth, and seed weight, reinforcing the impact of assisted gene flow on these traits, already observed in the phenotypic study conducted in the common garden experiment. Although we cannot demonstrate that the variations in these traits are due to the observed changes in these genomic areas, we present some evidence compatible with this idea, thus opening the door to explore these findings further.

Certain identified loci, such as YABBY 1‐like and xyloglucan endotransglucosylase/hydrolase protein, have previously been linked with floral development and abiotic stress responses (Kumaran et al. 2002; Siegfried et al. 1999; Maris et al. 2009; Keun et al. 2006; Nazari et al. 2020). The influence of assisted gene flow on the Flowering Locus T (FT) and EBS proteins is particularly noteworthy. FT is a key element in the induction of flowering in *Arabidopsis thaliana* and other species, such as legumes (e.g., Kardailsky et al. 1999; Pin and Nilsson 2012; Weller and Ortega 2015). The EBS protein regulates chromatin expression, controlling the expression of genes including FT (López‐González et al. 2014). Therefore, our study identifies FT and EBS as promising candidates for assisted gene flow management programs aimed at modifying flowering patterns. However, these results should be interpreted with caution, as this kind of approach is associated with possible false positives (Pavlidis et al. 2012), and spurious associations may be present (e.g., due to insufficiently strict linkage disequilibrium filtering). Furthermore, population structure may still confound the relationship between genetic variants and phenotypic effects. Therefore, further studies across independent populations would help validate these associations.

Despite the above‐mentioned limitations, this study is among the first to evaluate the use of assisted gene flow from both phenotypic and genomic perspectives. Our findings demonstrate that including genomic analyses in assisted gene flow studies offers more accurate information about genetically induced phenotypic changes. In addition, the identification of these genes paves the way for developing specific markers to identify early flowering genotypes in the species.

### 4.3 Final Conclusions

Gene flow, particularly assisted gene flow facilitated by human intervention, can enhance genetic diversity and contribute to population adaptation to climate change by introducing suitable genetic variation (Grummer et al. 2022). Our approach provides a novel framework where assisted gene flow could aid in the recovery of climate‐threatened populations. We found that assisted gene flow can modify key adaptive traits, such as flowering onset, potentially enhancing adaptive potential in warmer, drier environments.

However, this strategy is not without challenges. Unanticipated effects may occur in different traits, and the impacts of assisted gene flow will largely depend on the characteristics of donor and recipient populations. This strategy will be most effective when the source populations are previously adapted to the environmental conditions currently experienced by the target population (Aitken and Whitlock 2013; Prieto‐Benítez et al. 2021). To enable an appropriate assessment, genomic, phenotypic, and phenological information must be available and remain essential to design a successful assisted gene flow approach.

Despite these considerations, our proof‐of‐concept study using assisted gene flow suggests that this strategy holds promise for biodiversity conservation in the face of climate change. This study, one of the first of its kind, also demonstrates the potential of including genomic analyses to identify targeted regions and assess the real impact of the strategy on the genomes of the populations. Further research is needed, particularly studies conducted under natural conditions where selective pressures may differ, potentially affecting the performance of individuals from assisted gene flow lines.

## Conflicts of Interest

The authors declare no conflicts of interest.

## Supporting information


**Figure S1.** Effect of the gene flow line (GFL) on the different traits of *Lupinus angustifolius* L.: (a) number of seeds, (b) height, (c) biomass, (d) SLA, (e) LDMC. Dots and bars represent the predicted mean from the LMM model with a Gaussian distribution and 95% confidence intervals. Differences between the gene flow line and the control line were non‐significant.

**Figure S2.** Correlations between flowering onset and other plant traits for control line and year 2019. (a) Correlations for FRO population. (b) Correlations for PIC population. Positive correlations are represented in cold colors, while negative correlations are represented in warm colors. Non‐significant correlations (*p* > 0.05) are represented in grey.

**Figure S3.** Distribution of flowering onset (days) according to the genotypes (homozygous AA, aa and heterozygous Aa) for the 36 significant SNPs detected.

**Figure S4.** Distribution of seed weight (mg) according to the genotypes (homozygous AA, aa and heterozygous Aa) for the 36 significant SNPs detected.

**Figure S5.** Distribution of shoot growth (cm) according to the genotypes (homozygous AA, aa and heterozygous Aa) for the 36 significant SNPs detected.

**Figure S6.** Changes in allele frequencies between the control treatment (blue) and the gene flow treatment (purple) for both populations (FRO and PIC) for the 36 significant SNPs detected.

Table S1.

## Data Availability

Data associated with this study are made available in the figshare data repository: 10.6084/m9.figshare.28304024.
