# Supplementary figures and images for: Assisted Gene Flow Management to Climate Change in the Annual Legume Lupinus angustifolius L.: From Phenotype to Genotype
Source: Evol Appl. 2025 Mar 6;18(3):e70087. doi: 10.1111/eva.70087 (PMC11885171; doi:10.1111/eva.70087)

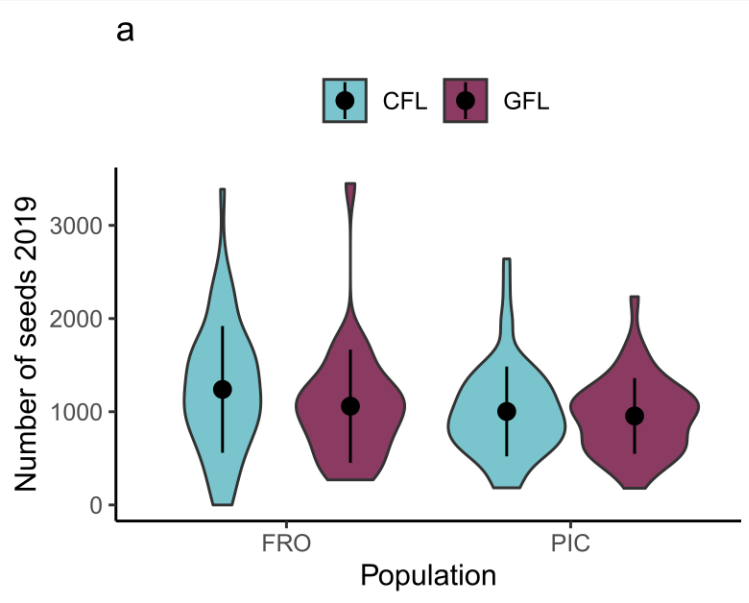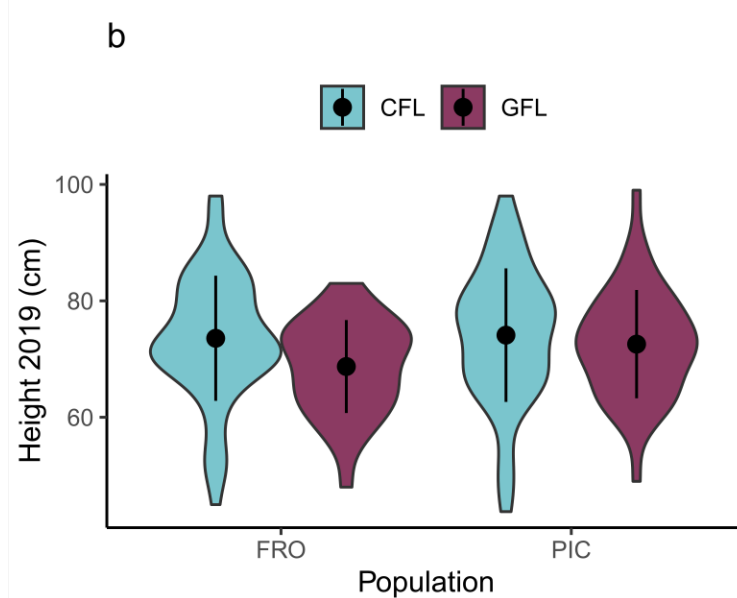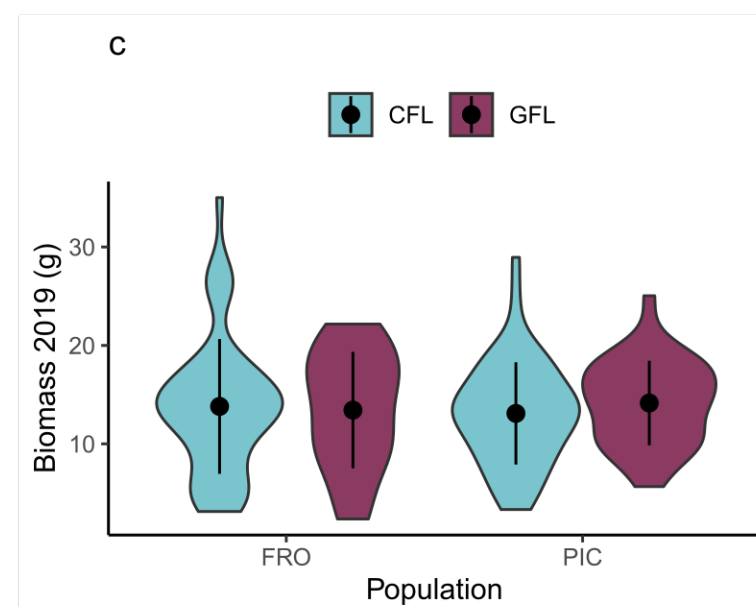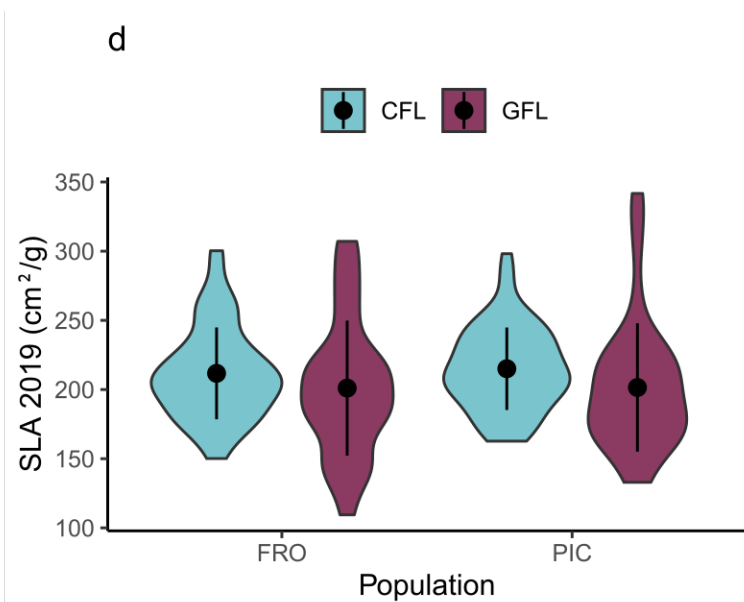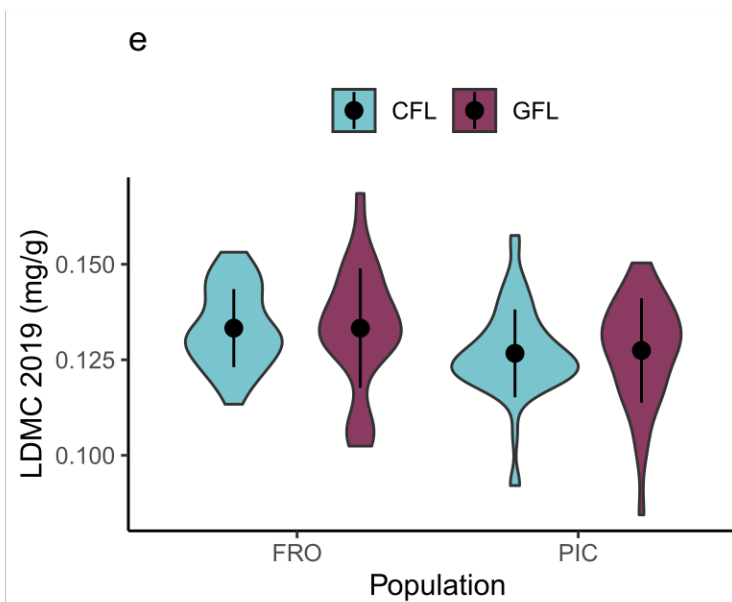

Supplement: Supplementary file 1 — Figure S1. Effect of the gene flow line (GFL) on the different traits of Lupinus angustifolius L.: (a) number of seeds, (b) height, (c) biomass, (d) SLA, (e) LDMC. Dots and bars represent the predicted mean from the LMM model with a Gaussian distribution and 95% confidence intervals. Differences between the gene flow line and the control line were non‐significant. [file EVA-18-e70087-s004.pdf]

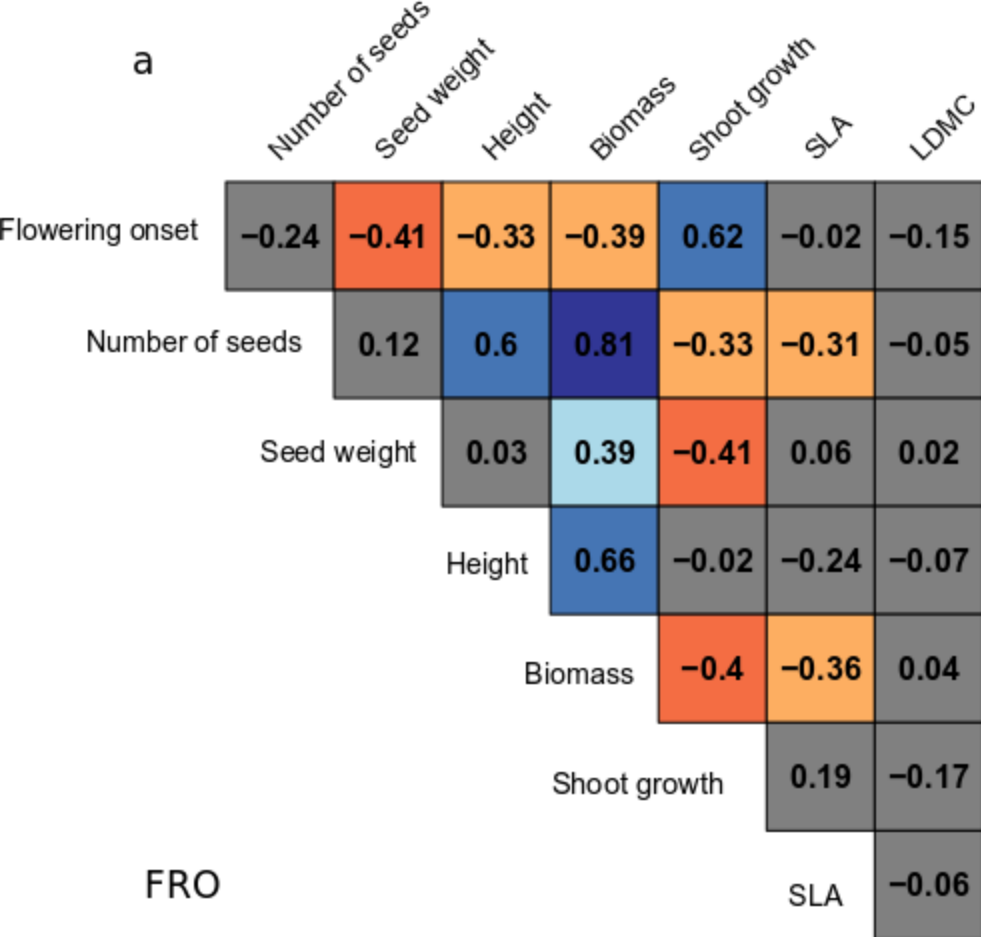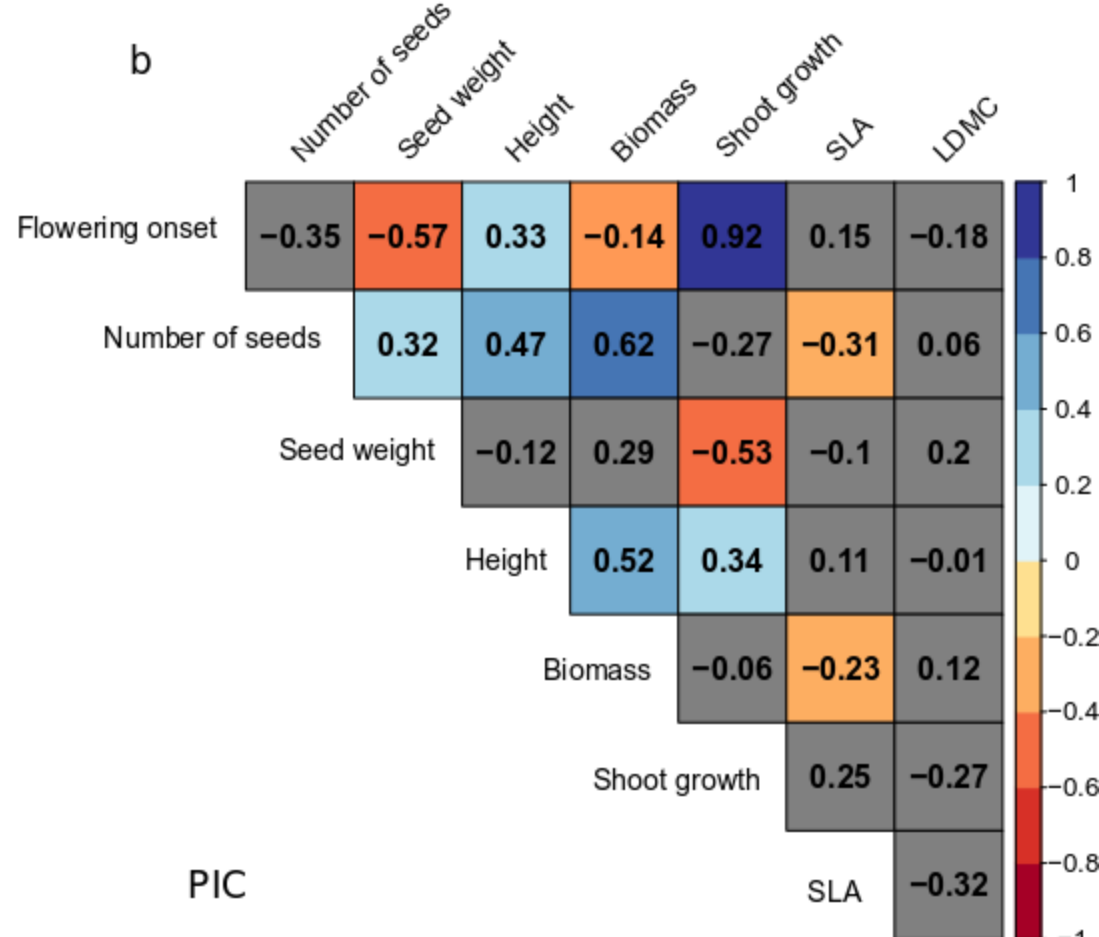

Supplement: Supplementary file 2 — Figure S2. Correlations between flowering onset and other plant traits for control line and year 2019. (a) Correlations for FRO population. (b) Correlations for PIC population. Positive correlations are represented in cold colors, while negative correlations are represented in warm colors. Non‐significant correlations (p > 0.05) are represented in grey. [file EVA-18-e70087-s006.pdf]

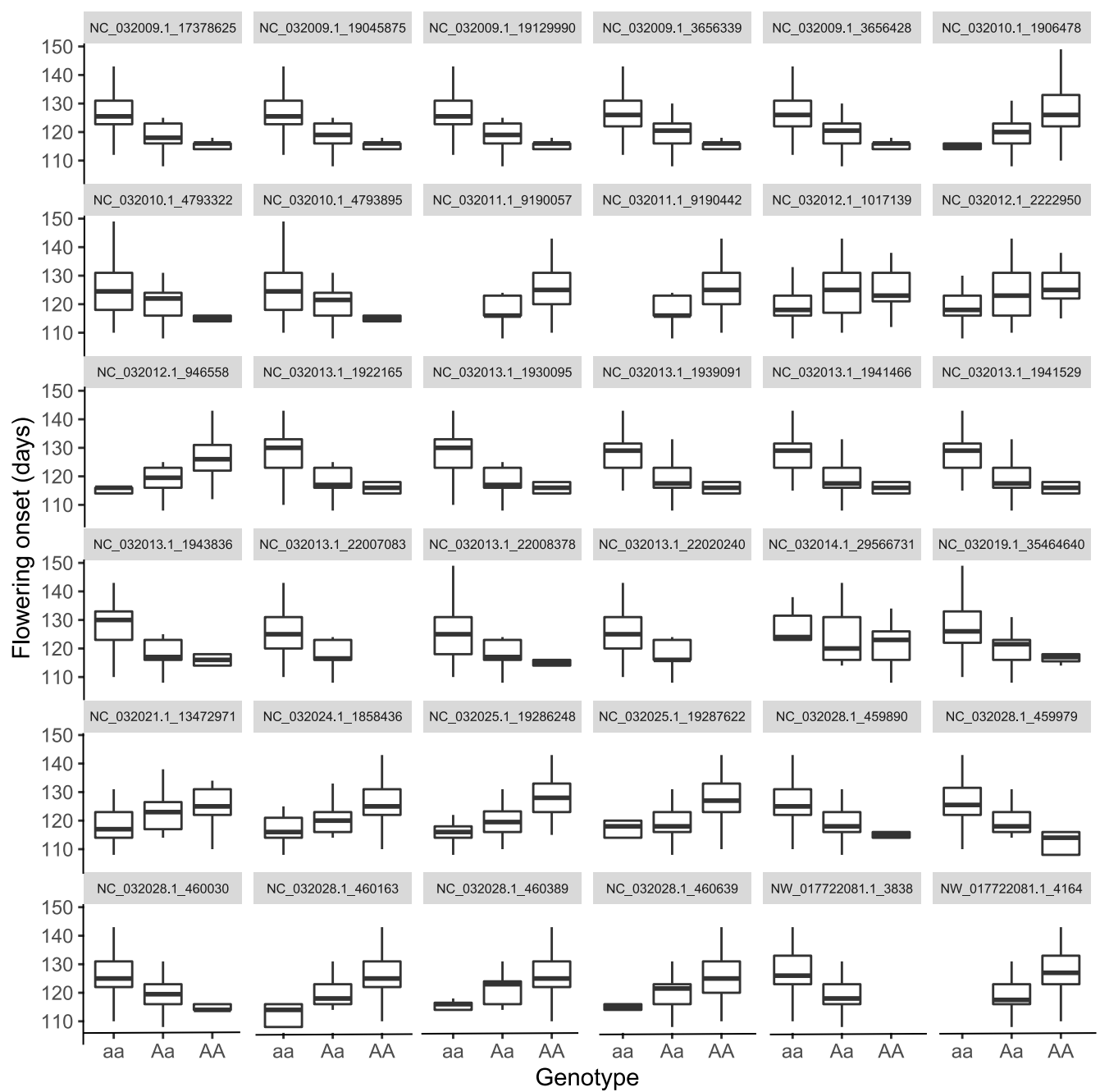

Supplement: Supplementary file 3 — Figure S3. Distribution of flowering onset (days) according to the genotypes (homozygous AA, aa and heterozygous Aa) for the 36 significant SNPs detected. [file EVA-18-e70087-s001.pdf]

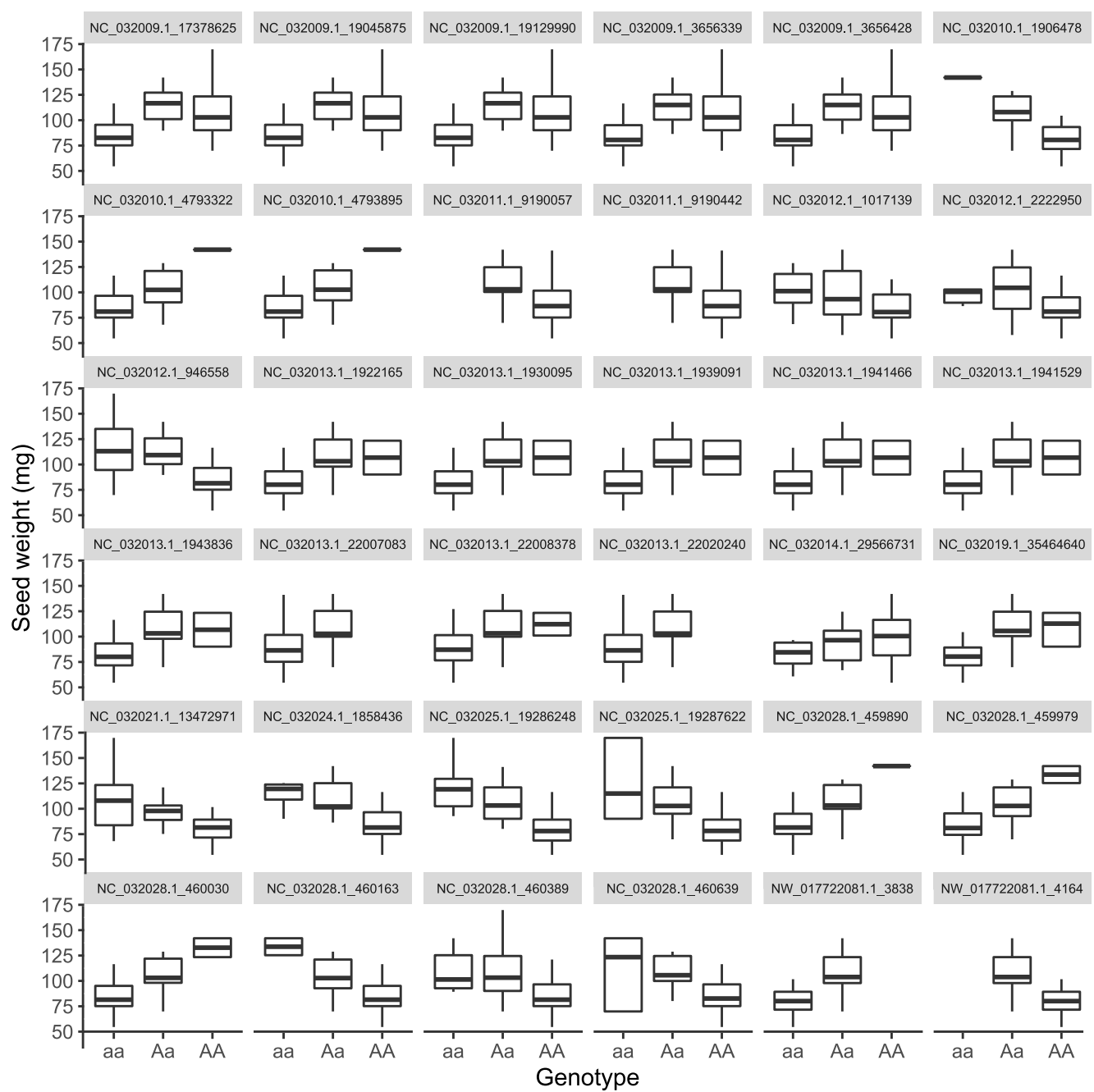

Supplement: Supplementary file 4 — Figure S4. Distribution of seed weight (mg) according to the genotypes (homozygous AA, aa and heterozygous Aa) for the 36 significant SNPs detected. [file EVA-18-e70087-s007.pdf]

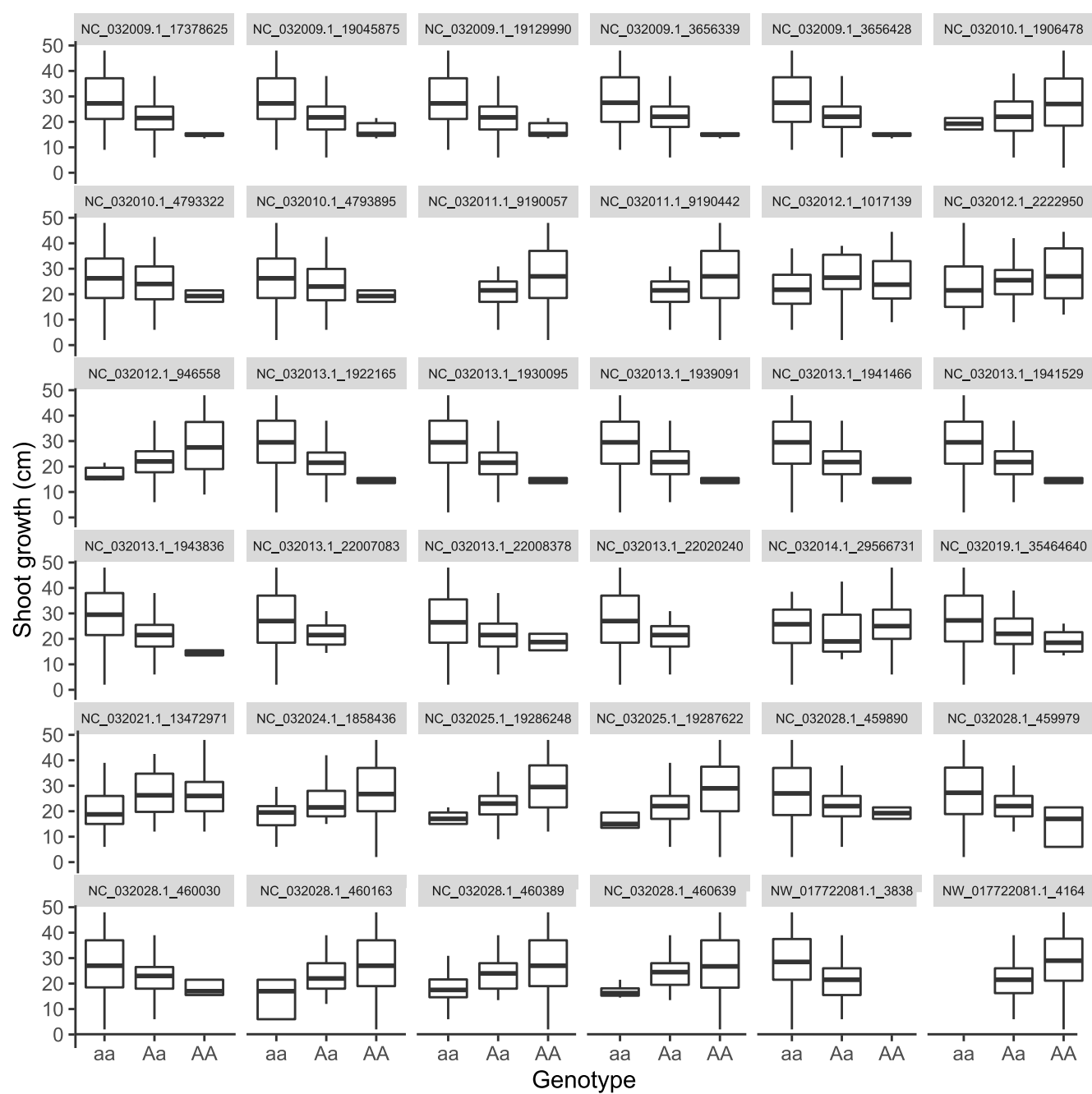

Supplement: Supplementary file 5 — Figure S5. Distribution of shoot growth (cm) according to the genotypes (homozygous AA, aa and heterozygous Aa) for the 36 significant SNPs detected. [file EVA-18-e70087-s002.pdf]

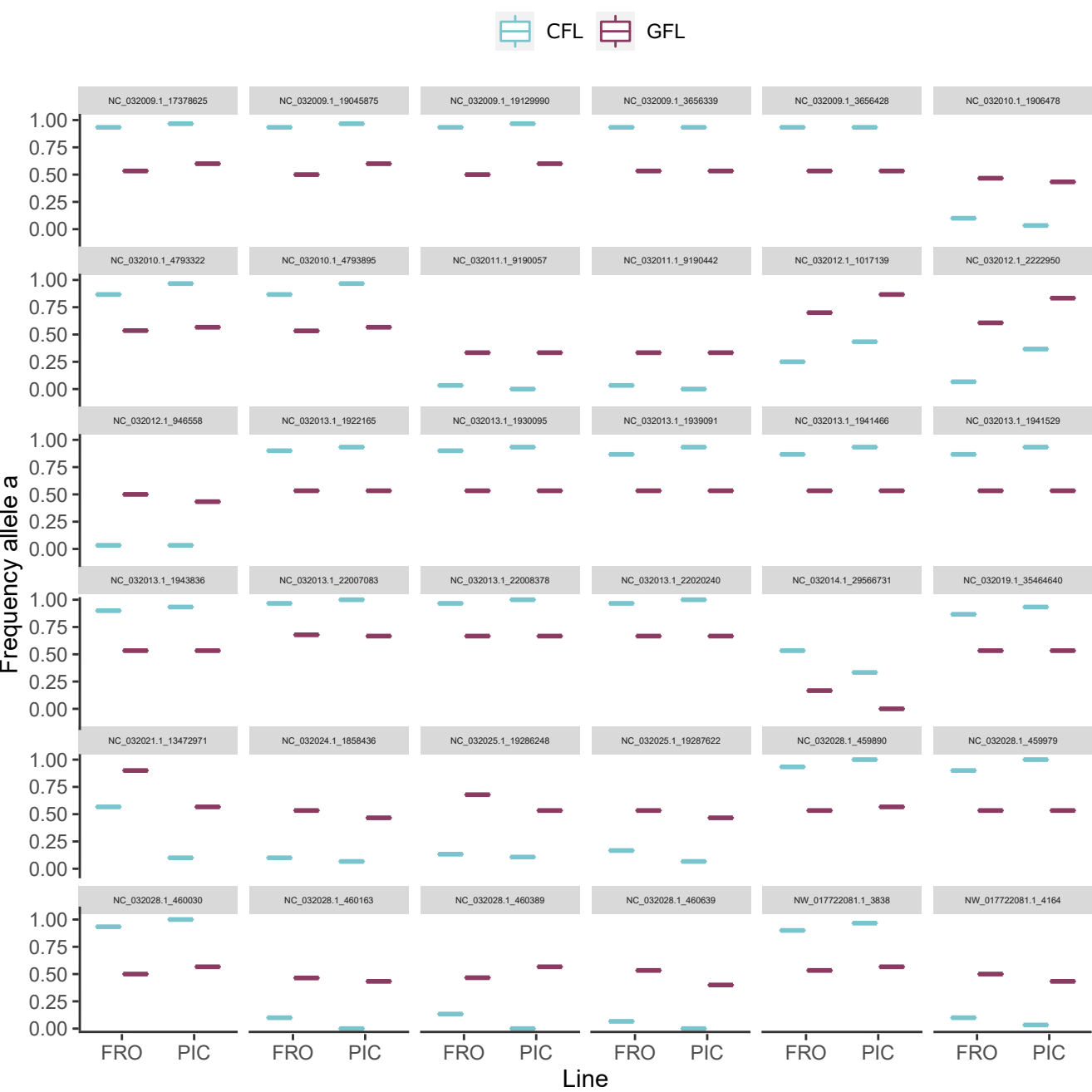

Supplement: Supplementary file 6 — Figure S6. Changes in allele frequencies between the control treatment (blue) and the gene flow treatment (purple) for both populations (FRO and PIC) for the 36 significant SNPs detected. [file EVA-18-e70087-s003.pdf]
