# Supplementary material for: Assisted Gene Flow Management to Climate Change in the Annual Legume Lupinus angustifolius L.: From Phenotype to Genotype
Source: Evol Appl. 2025 Mar 6;18(3):e70087. doi: 10.1111/eva.70087 (PMC11885171; doi:10.1111/eva.70087)
Supplement: Supplementary file 7 — Table S1. [file EVA-18-e70087-s005.docx]

**Supplementary Material**

Table S1. Observed mean ± SD values for the different traits measured and the different lines tested for each population. CFL: control line; GFL: F1 gene flow lines obtained by crossing FRO and PIC mother plants with GAR and RIV pollen, respectively; SPL: F2 self-pollination line; BCL: backcross line. SLA: specific leaflet area; LDMC: leaflet dry matter content.

|  | Flowering onset 2019 (days) | | Flowering onset  2020 (days) | | | Number of seeds | | Seed weight (mg) | | Height (cm) | | Biomass (g) | | Shoot growth (cm) | | SLA (cm^2^/g) | | LDMC (mg/g) | |
| --- | --- | --- | --- | --- | --- | --- | --- | --- | --- | --- | --- | --- | --- | --- | --- | --- | --- | --- | --- |
| Line | CFL | GFL | CFL | BCL | SPL | CFL | GFL | CFL | GFL | CFL | GFL | CFL | GFL | CFL | GFL | CFL | GFL | CFL | GFL |
| FRO | 127,38 ± 9,39 | 120,22  ±6,84 | 121,27  ±9,52 | 109,44  ±7,91 | 115,03  ±8,81 | 1239,46  ±679,73 | 1059,49  ±607,55 | 87,95  ±19,59 | 114,97  ±10,75 | 73,57  ±10,75 | 68,72  ±7,98 | 13,81  ±6,85 | 13,43  ±5,91 | 26,39  ±8,64 | 22,84  ±7,85 | 211,69  ±33,22 | 201,03  ±48,78 | 0,13  ±0,01 | 0,13  ±0,02 |
| PIC | 128,18 ± 9,65 | 120,02  ±6,98 | 119,98  ±13,12 | - | 113,24  ±8,22 | 1003,01  ±481,72 | 954,48  ±405,46 | 80,79  ±23,92 | 97,48  ±17,06 | 74,11  ±11,47 | 72,56  ±9,31 | 13,10  ±5,20 | 14,15  ±4,29 | 28,16  ±10,65 | 23,58  ±8,41 | 215,03  ±29,83 | 201,46  ±46,38 | 0,13  ±0,01 | 0,12  ±0,02 |
| GAR | 114,92 ± 6,81 | - | 109,73 ± 7,04 | - | - | 527,17  ± 393,35 | - | 130,41  ±28,18 | - | 65,70 ±9,99 | - | 13,10  ±5,49 | - | 15,50  ±7,72 | - | 201,72  ±35,18 | - | 0,12  ±0,01 | - |
| RIV | 109,10 ± 6,01 | - | 107,45 ± 7,14 | - | - | 607,78  ± 484,48 | - | 130,61  ±14,55 | - | 56,87 ±8,56 | - | 14,16  ±5,72 | - | 9,41  ±4,98 | - | 175,31  ±27,01 | - | 0,13  ±0,01 | - |

Table S2. Effect of the different lines and population on flowering onset, number of seeds, seed weight, height, biomass, shoot growth, SLA and LDMC of *Lupinus angustifolius* plants in the common garden experiment. Estimates and significance level for fixed effects are shown. Genotype was included as a random factor. CFL: control line; GFL: F1 gene flow line; SPL: F2 self-pollination line; BCL: backcross line. Missing factors (CFL = Control line and population FRO) are included in the intercept.

| Fixed effects | Parameter value | Standard error | z/t value | p value | Pseudo-R^2^ (fixed effects) | Pseudo- R^2^ (total) |
| --- | --- | --- | --- | --- | --- | --- |
| Flowering onset 2019 | - | - | - | - | 0.122 | 0.140 |
| Intercept | 4.920 | 0.036 | 147.861 | <0.001 | - | - |
| GFL | -0.058 | 0.014 | -4.174 | <0.001 | - | - |
| PIC | -0.003 | 0.013 | -0.200 | 0.841 | - | - |
| Flowering onset 2020 | - | - | - | - | 0.166 | 0.265 |
| Intercept | 4.917 | 0.037 | 132.799 | <0.001 | - | - |
| BCL | -0.078 | 0.034 | -2.296 | 0.022 | - | - |
| SPL | -0.033 | 0.018 | -1.808 | 0.071 | - | - |
| PIC | -0.020 | 0.016 | -1.189 | 0.235 | - | - |
| Number of seeds | - | - | - | - | 0.068 | 0.137 |
| Intercept | 738.358 | 208.134 | 112.053 | <0.001 | - | - |
| GFL | -133.430 | 90.330 | 51.862 | 0.146 | - | - |
| PIC | -148.332 | 86.265 | 85.025 | 0.089 | - | - |
| Seed weight | - | - | - | - | 0.310 | 0.466 |
| Intercept | 51.621 | 8.597 | 6.004 | <0.001 | - | - |
| GFL | 19.519 | 3.882 | 5.028 | <0.001 | - | - |
| PIC | -6.408 | 3.523 | -1.819 | 0.072 | - | - |
| Height | - | - | - | - | 0.022 | 0.197 |
| Intercept | 70.444 | 4.110 | 17.140 | <0.001 | - | - |
| GFL | -2.948 | 1.868 | -1.578 | 0.121 | - | - |
| PIC | 1.417 | 1.721 | 0.823 | 0.413 | - | - |
| Biomass | - | - | - | - | 0.091 | 0.177 |
| Intercept | 4.869 | 2.378 | 2.047 | 0.043 | - | - |
| GFL | 0.165 | 0.975 | 0.169 | 0.866 | - | - |
| PIC | 0.621 | 0.944 | 0.658 | 0.512 | - | - |
| Shoot growth | - | - | - | - | 0.103 | 0.505 |
| Intercept | 38.298 | 3.567 | 10.735 | <0.001 | - | - |
| GFL | -3.677 | 1.975 | -1.861 | 0.068 | - | - |
| PIC | -1.240 | 1.632 | -0.760 | 0.449 | - | - |
| SLA | - | - | - | - | 0.047 | 0.150 |
| Intercept | 240.312 | 14.440 | 16.642 | <0.001 | - | - |
| GFL | -10.589 | 6.345 | -1.669 | 0.102 | - | - |
| PIC | -0.543 | 5.925 | -0.092 | 0.927 | - | - |
| LDMC | - | - | - | - | 0.072 | 0.074 |
| Intercept | 0.127 | 0.005 | 28.408 | <0.001 | - | - |
| GFL | -0.000 | 0.002 | -0.030 | 0.976 | - | - |
| PIC | -0.006 | 0.002 | -3.240 | 0.002 | - | - |

Table S3. Chi-square statistic, degrees of freedom and p-values of the Type II Wald chi-square tests of GLMM and LMM analyses to study the effect of selection line, population, and year on flowering onset, number of seeds, seed weight, height, biomass, shoot growth, SLA and LDMC of *Lupinus angustifolius* plants grown in the common garden experiment. Estimates and significance level for fixed effects are shown. Genotype was included as a random factor.

| Fixed effects | *X*^2^ | Df | Pr (>\|*X*^2^\|) |
| --- | --- | --- | --- |
| Flowering onset 2019 | - | - | - |
| Line | 17.422 | 1 | <0.001 |
| Population | 0.040 | 1 | 0.841 |
| Flowering onset 2020 | - | - | - |
| Line | 6.958 | 2 | 0.031 |
| Population | 1.413 | 1 | 0.235 |
| Number of seeds | - | - | - |
| Line | 2.181 | 1 | 0.140 |
| Population | 2.957 | 1 | 0.086 |
| Seed weight | - | - | - |
| Line | 25.280 | 1 | <0.001 |
| Population | 3.309 | 1 | 0.069 |
| Height | - | - | - |
| Line | 2.490 | 1 | 0.115 |
| Population | 0.678 | 1 | 0.410 |
| Biomass | - | - | - |
| Line | 0.029 | 1 | 0.866 |
| Population | 0.433 | 1 | 0.510 |
| Shoot growth | - | - | - |
| Line | 3.464 | 1 | 0.063 |
| Population | 0.578 | 1 | 0.447 |
| SLA | - | - | - |
| Line | 2.785 | 1 | 0.095 |
| Population | 0.008 | 1 | 0.927 |
| LDMC | - | - | - |
| Line | 0.000 | 1 | 0.976 |
| Population | 10.495 | 1 | 0.001 |

Table S4. Posterior mean values, standard errors and 95 % confidence intervals for the different traits and lines of *Lupinus angustifolius* plants grown in a common garden experiment. CFL: control line; GFL: F1 gene flow line; SPL: F2 self-pollination line; BCL: backcross line.

|  | FRO | | | | PIC | | | |
| --- | --- | --- | --- | --- | --- | --- | --- | --- |
|  | **Mean** | **Std. error** | **2∙5%** | **97∙%** | **Mean** | **Std. error** | **2∙5%** | **97∙%** |
| Flowering onset 2019 | - | - | - | - | - | - | - | - |
| CFL | 128 | 1.34 | 125 | 130 | 127 | 1.37 | 125 | 130 |
| GFL | 121 | 1.63 | 117 | 124 | 120 | 1.49 | 117 | 123 |
| Flowering onset 2020 | - | - | - | - | - | - | - | - |
| CFL | 120 | 1.70 | 117 | 124 | 118 | 1.73 | 115 | 121 |
| BCL | 111 | 3.44 | 105 | 118 | 109 | 3.81 | 102 | 117 |
| SPL | 116 | 1.88 | 113 | 120 | 114 | 1.64 | 111 | 117 |
| Number of seeds | - | - | - | - | - | - | - | - |
| CFL | 1201 | 70.10 | 1061 | 1341 | 1053 | 70.10 | 913 | 1193 |
| GFL | 1068 | 87.70 | 892 | 1244 | 919 | 79.40 | 760 | 1079 |
| Seed weight | - | - | - | - | - | - | - | - |
| CFL | 87.80 | 2.90 | 82.0 | 93.60 | 81.40 | 2.93 | 75.60 | 87.20 |
| GFL | 107.30 | 3.76 | 99.8 | 114.90 | 100.90 | 3.39 | 94.10 | 107.70 |
| Height | - | - | - | - | - | - | - | - |
| CFL | 73.00 | 1.40 | 70.20 | 75.80 | 74.40 | 1.41 | 71.60 | 77.20 |
| GFL | 70.00 | 1.81 | 66.40 | 73.60 | 71.40 | 1.66 | 68.10 | 74.80 |
| Biomass | - | - | - | - | - | - | - | - |
| CFL | 13.20 | 0.75 | 11.70 | 14.70 | 13.80 | 0.76 | 12.30 | 15.30 |
| GFL | 13.30 | 0.96 | 11.40 | 15.20 | 13.90 | 0.87 | 12.20 | 15.70 |
| Shoot growth | - | - | - | - | - | - | - | - |
| CFL | 27.80 | 1.40 | 25.00 | 30.60 | 26.60 | 1.42 | 23.80 | 29.40 |
| GFL | 24.10 | 1.87 | 20.40 | 27.90 | 22.90 | 1.74 | 19.40 | 26.40 |
| SLA | - | - | - | - | - | - | - | - |
| CFL | 213 | 4.76 | 203 | 222 | 212 | 4.85 | 203 | 222 |
| GFL | 202 | 6.14 | 190 | 215 | 202 | 5.64 | 191 | 213 |
| LDMC | - | - | - | - | - | - | - | - |
| CFL | 0.13 | 0.00 | 0.13 | 0.14 | 0.13 | 0.00 | 0.12 | 0.13 |
| GFL | 0.13 | 0.00 | 0.13 | 0.14 | 0.13 | 0.00 | 0.12 | 0.13 |

Table S5. Minimum allele frequency (MAF), False Discovery Rate (FDR), F_ST_ statistic, F_ST_-FDR, and e-value for each SNP identified as outlier in the genomic analyses.

|  |  |  |  |  |  |  |  | CFL | GFL |
| --- | --- | --- | --- | --- | --- | --- | --- | --- | --- |
| Population | **SNP** | **Gene** | **Protein** | **FDR** | **F_ST_** | **F_ST_-FDR** | **e-value** | **MAF** | **MAF** |
| FRO | NC_032009.1_17378625 | LOC109350320 | XP_019447099.1 | 0.010 | 0.313 | <0.001 | 5e-145 | 0.067 | 0.467 |
| FRO | NC_032009.1_19045875 | LOC109351227 | XP_019448173.1 | 0.006 | 0.348 | <0.001 | 2e-180 | 0.067 | 0.500 |
| FRO | NC_032009.1_19129990 | LOC109351285 | XP_019448262.1 | 0.006 | 0.348 | <0.001 | 0 | 0.067 | 0.500 |
| FRO | NC_032009.1_3656339 | LOC109347866 | XP_019443510.1 | 0.010 | 0.313 | <0.001 | 0 | 0.067 | 0.467 |
| FRO | NC_032009.1_3656428 | LOC109347866 | XP_019443510.1 | 0.010 | 0.313 | <0.001 | 0 | 0.067 | 0.467 |
| FRO | NC_032010.1_1906478 | LOC109331801 | XP_019422070.1 | 0.023 | 0.266 | 0.001 | 4e-131 | 0.100 | 0.467 |
| FRO | NC_032010.1_4793322 | LOC109328838 | XP_019417994.1 | 0.045 | 0.212 | 0.003 | 0 | 0.133 | 0.464 |
| FRO | NC_032010.1_4793895 | LOC109328838 | XP_019417994.1 | 0.048 | 0.214 | 0.003 | 0 | 0.133 | 0.467 |
| FRO | NC_032011.1_9190057 | LOC109343327 | XP_019437115.1 | 0.033 | 0.247 | 0.001 | 0 | 0.033 | 0.333 |
| FRO | NC_032011.1_9190442 | LOC109343327 | XP_019437115.1 | 0.033 | 0.247 | 0.001 | 0 | 0.033 | 0.333 |
| FRO | NC_032012.1_1017139 | LOC109345004 | XP_019439298.1 | 0.009 | 0.309 | <0.001 | 0 | 0.250 | 0.700 |
| FRO | NC_032012.1_2222950 | LOC109345069 | XP_019439392.1 | 0.001 | 0.489 | <0.001 | 0 | 0.067 | 0.607 |
| FRO | NC_032012.1_946558 | LOC109344999 | XP_019439290.1 | 0.002 | 0.410 | <0.001 | 0 | 0.033 | 0.500 |
| FRO | NC_032013.1_1922165 | LOC109347401 | XP_019442778.1 | 0.023 | 0.271 | 0.001 | 0 | 0.100 | 0.467 |
| FRO | NC_032013.1_1930095 | LOC109347401 | XP_019442778.1 | 0.023 | 0.271 | 0.001 | 0 | 0.100 | 0.467 |
| FRO | NC_032013.1_1939091 | LOC109347400 | XP_019442777.1 | 0.048 | 0.219 | 0.002 | 0 | 0.133 | 0.467 |
| FRO | NC_032013.1_1941466 | LOC109347400 | XP_019442777.1 | 0.048 | 0.219 | 0.002 | 0 | 0.133 | 0.467 |
| FRO | NC_032013.1_1941529 | LOC109347400 | XP_019442777.1 | 0.048 | 0.219 | 0.002 | 0 | 0.133 | 0.467 |
| FRO | NC_032013.1_1943836 | LOC109347400 | XP_019442777.1 | 0.023 | 0.271 | 0.001 | 0 | 0.100 | 0.467 |
| FRO | NC_032013.1_22007083 | LOC109348120 | XP_019443898.1 | 0.029 | 0.238 | 0.001 | 0 | 0.033 | 0.321 |
| FRO | NC_032013.1_22008378 | LOC109348120 | XP_019443898.1 | 0.033 | 0.240 | 0.001 | 0 | 0.033 | 0.333 |
| FRO | NC_032013.1_22020240 | LOC109348121 | XP_019443899.1 | 0.033 | 0.247 | 0.001 | 7e-158 | 0.033 | 0.333 |
| FRO | NC_032014.1_29566731 | LOC109350790 | XP_019447641.1 | 0.035 | 0.229 | 0.002 | 0 | 0.467 | 0.833 |
| FRO | NC_032019.1_35464640 | LOC109360702 | XP_019461304.1 | 0.048 | 0.209 | 0.003 | 0 | 0.133 | 0.467 |
| FRO | NC_032021.1_13472971 | LOC109325863 | XP_019414003.1 | 0.040 | 0.227 | 0.002 | 0 | 0.433 | 0.100 |
| FRO | NC_032024.1_1858436 | LOC109329933 | XP_019419387.1 | 0.008 | 0.326 | <0.001 | 0 | 0.100 | 0.533 |
| FRO | NC_032025.1_19286248 | LOC109331033 | XP_019420861.1 | 0.002 | 0.457 | <0.001 | 2e-126 | 0.133 | 0.679 |
| FRO | NC_032025.1_19287622 | LOC109331033 | XP_019420861.1 | 0.035 | 0.243 | 0.001 | 2e-126 | 0.167 | 0.533 |
| FRO | NC_032028.1_459890 | LOC109335596 | XP_019427286.1 | 0.01 | 0.323 | <0.001 | 0 | 0.067 | 0.467 |
| FRO | NC_032028.1_459979 | LOC109335596 | XP_019427286.1 | 0.023 | 0.266 | 0.001 | 0 | 0.100 | 0.467 |
| FRO | NC_032028.1_460030 | LOC109335596 | XP_019427286.1 | 0.006 | 0.357 | <0.001 | 0 | 0.067 | 0.500 |
| FRO | NC_032028.1_460163 | LOC109335596 | XP_019427286.1 | 0.021 | 0.265 | 0.001 | 0 | 0.100 | 0.464 |
| FRO | NC_032028.1_460389 | LOC109335596 | XP_019427286.1 | 0.048 | 0.204 | 0.003 | 0 | 0.133 | 0.467 |
| FRO | NC_032028.1_460639 | LOC109335596 | XP_019427286.1 | 0.003 | 0.392 | <0.001 | 0 | 0.067 | 0.533 |
| FRO | NW_017722081.1_3838 | LOC109338905 | XP_019431800.1 | 0.023 | 0.276 | 0.001 | 0 | 0.100 | 0.467 |
| FRO | NW_017722081.1_4164 | LOC109338905 | XP_019431800.1 | 0.013 | 0.314 | <0.001 | 0 | 0.100 | 0.500 |
| PIC | NC_032009.1_17378625 | LOC109350320 | XP_019447099.1 | 0.045 | 0.315 | 0.001 | 5e-145 | 0.033 | 0.400 |
| PIC | NC_032009.1_19045875 | LOC109351227 | XP_019448173.1 | 0.045 | 0.315 | 0.001 | 2e-180 | 0.033 | 0.400 |
| PIC | NC_032009.1_19129990 | LOC109351285 | XP_019448262.1 | 0.045 | 0.315 | 0.001 | 0 | 0.033 | 0.400 |
| PIC | NC_032009.1_3656339 | LOC109347866 | XP_019443510.1 | 0.040 | 0.328 | 0.001 | 0 | 0.067 | 0.467 |
| PIC | NC_032009.1_3656428 | LOC109347866 | XP_019443510.1 | 0.040 | 0.328 | 0.001 | 0 | 0.067 | 0.467 |
| PIC | NC_032010.1_1906478 | LOC109331801 | XP_019422070.1 | 0.029 | 0.358 | <0.001 | 4e-131 | 0.033 | 0.433 |
| PIC | NC_032010.1_4793322 | LOC109328838 | XP_019417994.1 | 0.029 | 0.358 | <0.001 | 0 | 0.033 | 0.433 |
| PIC | NC_032010.1_4793895 | LOC109328838 | XP_019417994.1 | 0.029 | 0.358 | <0.001 | 0 | 0.033 | 0.433 |
| PIC | NC_032011.1_9190057 | LOC109343327 | XP_019437115.1 | 0.040 | 0.321 | 0.001 | 0 | <0.001 | 0.333 |
| PIC | NC_032011.1_9190442 | LOC109343327 | XP_019437115.1 | 0.040 | 0.321 | 0.001 | 0 | <0.001 | 0.333 |
| PIC | NC_032012.1_1017139 | LOC109345004 | XP_019439298.1 | 0.040 | 0.315 | 0.001 | 0 | 0.433 | 0.867 |
| PIC | NC_032012.1_2222950 | LOC109345069 | XP_019439392.1 | 0.032 | 0.349 | <0.001 | 0 | 0.367 | 0.833 |
| PIC | NC_032012.1_946558 | LOC109344999 | XP_019439290.1 | 0.029 | 0.358 | <0.001 | 0 | 0.033 | 0.433 |
| PIC | NC_032013.1_1922165 | LOC109347401 | XP_019442778.1 | 0.040 | 0.328 | 0.001 | 0 | 0.067 | 0.467 |
| PIC | NC_032013.1_1930095 | LOC109347401 | XP_019442778.1 | 0.040 | 0.328 | 0.001 | 0 | 0.067 | 0.467 |
| PIC | NC_032013.1_1939091 | LOC109347400 | XP_019442777.1 | 0.040 | 0.328 | 0.001 | 0 | 0.067 | 0.467 |
| PIC | NC_032013.1_1941466 | LOC109347400 | XP_019442777.1 | 0.040 | 0.328 | 0.001 | 0 | 0.067 | 0.467 |
| PIC | NC_032013.1_1941529 | LOC109347400 | XP_019442777.1 | 0.040 | 0.328 | 0.001 | 0 | 0.067 | 0.467 |
| PIC | NC_032013.1_1943836 | LOC109347400 | XP_019442777.1 | 0.040 | 0.328 | 0.001 | 0 | 0.067 | 0.467 |
| PIC | NC_032013.1_22007083 | LOC109348120 | XP_019443898.1 | 0.040 | 0.321 | 0.001 | 0 | <0.001 | 0.333 |
| PIC | NC_032013.1_22008378 | LOC109348120 | XP_019443898.1 | 0.040 | 0.314 | 0.001 | 0 | <0.001 | 0.333 |
| PIC | NC_032013.1_22020240 | LOC109348121 | XP_019443899.1 | 0.040 | 0.321 | 0.001 | 7e-158 | <0.001 | 0.333 |
| PIC | NC_032014.1_29566731 | LOC109350790 | XP_019447641.1 | 0.040 | 0.300 | 0.001 | 0 | 0.667 | 1 |
| PIC | NC_032019.1_35464640 | LOC109360702 | XP_019461304.1 | 0.040 | 0.328 | 0.001 | 0 | 0.067 | 0.467 |
| PIC | NC_032021.1_13472971 | LOC109325863 | XP_019414003.1 | 0.029 | 0.385 | <0.001 | 0 | 0.900 | 0.433 |
| PIC | NC_032024.1_1858436 | LOC109329933 | XP_019419387.1 | 0.040 | 0.318 | 0.001 | 0 | 0.067 | 0.467 |
| PIC | NC_032025.1_19286248 | LOC109331033 | XP_019420861.1 | 0.040 | 0.324 | 0.001 | 2e-126 | 0.107 | 0.533 |
| PIC | NC_032025.1_19287622 | LOC109331033 | XP_019420861.1 | 0.040 | 0.328 | 0.001 | 2e-126 | 0.067 | 0.467 |
| PIC | NC_032028.1_459890 | LOC109335596 | XP_019427286.1 | 0.009 | 0.429 | <0.001 | 0 | <0.001 | 0.433 |
| PIC | NC_032028.1_459979 | LOC109335596 | XP_019427286.1 | 0.009 | 0.459 | <0.001 | 0 | <0.001 | 0.467 |
| PIC | NC_032028.1_460030 | LOC109335596 | XP_019427286.1 | 0.009 | 0.429 | <0.001 | 0 | <0.001 | 0.433 |
| PIC | NC_032028.1_460163 | LOC109335596 | XP_019427286.1 | 0.009 | 0.423 | <0.001 | 0 | <0.001 | 0.433 |
| PIC | NC_032028.1_460389 | LOC109335596 | XP_019427286.1 | 0.005 | 0.555 | <0.001 | 0 | <0.001 | 0.567 |
| PIC | NC_032028.1_460639 | LOC109335596 | XP_019427286.1 | 0.021 | 0.393 | <0.001 | 0 | <0.001 | 0.400 |
| PIC | NW_017722081.1_3838 | LOC109338905 | XP_019431800.1 | 0.029 | 0.358 | <0.001 | 0 | 0.033 | 0.433 |
| PIC | NW_017722081.1_4164 | LOC109338905 | XP_019431800.1 | 0.029 | 0.358 | <0.001 | 0 | 0.033 | 0.433 |

Table S6. Functional annotation of the 36 SNPs identified as outliers in the genomic analyses.

| Name | Protein name | Protein ID | GO biological process |
| --- | --- | --- | --- |
| NC_032009.1_17378625 | axial regulator YABBY 1-like | XP_019447099.1 | Flower development (flowering) |
| NC_032009.1_19045875 | Uncharacterized protein | XP_019448173.1 | Meiotic nuclear division (reproduction) |
| NC_032009.1_19129990 | xyloglucanendotransglucosylase/hydrolaseprotein 28 | XP_019448262 | Stamen filament development (flowering) |
| NC_032009.1_3656339 | 3-oxoacyl-[acyl-carrier-protein] synthase II, chloroplastic-like | XP_019443510 | Response to cold (response to abiotic stress) |
| NC_032009.1_3656428 | 3-oxoacyl-[acyl-carrier-protein] synthase II, chloroplastic-like | XP_019443510.1 | Response to cold (response to abiotic stress) |
| NC_032010.1_1906478 | nitrogen regulatory protein P-II homolog | XP_019422070.1 | Regulation of nitrogen utilization (nitrogen) |
| NC_032010.1_4793322 | rop guanine nucleotide exchange factor 12-like | XP_019417994.1 | Pollentubegrowth (flowering) |
| NC_032010.1_4793895 | rop guanine nucleotide exchange factor 12-like | XP_019417994.1 | Pollentubegrowth (flowering) |
| NC_032011.1_9190057 | chaperone proteindnaJ GFA2, mitochondrial-likeisoform X3 | XP_019437115.1 | Pollination (reproduction) |
| NC_032011.1_9190442 | chaperone proteindnaJ GFA2, mitochondrial-likeisoform X3 | XP_019437115.1 | Pollination (reproduction) |
| NC_032012.1_1017139 | isoleucine--tRNA ligase, chloroplastic/mitochondrial | XP_019439298.1 | Ovule development (flowering) |
| NC_032012.1_2222950 | cytochrome P450 90A1-like | XP_019439392.1 | Anther differentiation (flowering) |
| NC_032012.1_946558 | protein pleiotropic regulatory locus 1-like | XP_019439290.1 | Cotyledon development (reproduction) |
| NC_032013.1_1922165 | cell division cycle protein 27 homolog B-like isoform X1 | XP_019442778.1 | Root meristem specification (growth) |
| NC_032013.1_1930095 | cell division cycle protein 27 homolog B-like isoform X1 | XP_019442778.1 | Root meristem specification (growth) |
| NC_032013.1_1939091 | cell division cycle protein 27 homolog B-like | XP_019442777.1 | Root meristem specification (growth) |
| NC_032013.1_1941466 | cell division cycle protein 27 homolog B-like | XP_019442777.1 | Root meristem specification (growth) |
| NC_032013.1_1941529 | cell division cycle protein 27 homolog B-like | XP_019442777.1 | Root meristem specification (growth) |
| NC_032013.1_1943836 | cell division cycle protein 27 homolog B-like | XP_019442777.1 | Root meristem specification (growth) |
| NC_032013.1_22007083 | chromatin remodeling protein EBS-like | XP_019443898.1 | Regulation of long-day photoperiodism (flowering) |
| NC_032013.1_22008378 | chromatin remodeling protein EBS-like | XP_019443898.1 | Regulation of long-day photoperiodism (flowering) |
| NC_032013.1_22020240 | chromatin remodeling protein EBS-like | XP_019443899.1 | Regulation of long-day photoperiodism (flowering) |
| NC_032014.1_29566731 | Cyclic nucleotide-binding domain-containing protein; putative cyclic nucleotide-gated ion channel 8 isoform X2 | OIW09331.1; XP_019447642.1 | Pollen tube growth (flowering) |
| NC_032019.1_35464640 | ubiquitin protein ligase | OIW01082.1 | Chromatin organization (NA) |
| NC_032021.1_13472971 | armadillo repeat-containing protein LFR | XP_019414003.1 | Anther development (flowering) |
| NC_032024.1_1858436 | nuclear export mediator factor NEMF | XP_019419388.1 | Cold acclimation (response to abiotic stress) |
| NC_032025.1_19286248 | protein FLOWERING LOCUS T-like | OIV93971 1; XP_019420861 | Response to short-day photoperiodism (flowering) |
| NC_032025.1_19287622 | protein FLOWERING LOCUS T-like | XP_019420861.1 | Response to short-day photoperiodism (flowering) |
| NC_032028.1_459890 | transcription factor RF2b-like | OIV91432.1; XP_019427286.1 | Response to sulfate (response to abiotic stress) |
| NC_032028.1_459979 | transcription factor RF2b-like | OIV91432.1; XP_019427286.2 | Response to sulfate (response to abiotic stress) |
| NC_032028.1_460030 | transcription factor RF2b-like | OIV91432.1; XP_019427286.3 | Response to sulfate (response to abiotic stress) |
| NC_032028.1_460163 | transcription factor RF2b-like | OIV91432.1; XP_019427286.4 | Response to sulfate (response to abiotic stress) |
| NC_032028.1_460389 | transcription factor RF2b-like | OIV91432.1; XP_019427286.5 | Response to sulfate (response to abiotic stress) |
| NC_032028.1_460639 | transcription factor RF2b-like | XP_019427286.1 | Response to sulfate (response to abiotic stress) |
| NW_017722081.1_3838 | ER lumen protein-retaining receptor | XP_019431801.1_1 | Meiotic nuclear division (reproduction) |
| NW_017722081.1_4164 | ER lumen protein-retaining receptor | XP_019431801.1_1 | Meiotic nuclear division (reproduction) |
